# Study on the role and mechanism of magnesium-calcium-mineralized collagen composite scaffolds in the adjuvant treatment of cervical cancer

**DOI:** 10.1093/rb/rbaf110

**Published:** 2025-11-12

**Authors:** Xiaojing Nie, Lei Wang, Wang Yang, Muyesier Maimaiti, Jingxin Yang, Chen Lin

**Affiliations:** School of Public Health, Xinjiang Medical University, Urumqi, Xinjiang 830000, P.R. China; Department of Pathology, School of Basic Medical Sciences, Xinjiang Medical University, Urumqi, Xinjiang 830000, P.R. China; Institute of Medical Sciences of Xinjiang Medical University, Urumqi, Xinjiang 830000, P.R.China; School of Public Health, Xinjiang Medical University, Urumqi, Xinjiang 830000, P.R. China; Department of Pathology, School of Basic Medical Sciences, Xinjiang Medical University, Urumqi, Xinjiang 830000, P.R. China; Pathology Department of Xinjiang Production, Construction Corps Hospital, Urumqi, Xinjiang 830000, P.R. China; Beijing Engineering Research Center of Smart Mechanical Innovation Design Service, Beijing Union University, Beijing 100027, P.R. China; Department of Pathology, School of Basic Medical Sciences, Xinjiang Medical University, Urumqi, Xinjiang 830000, P.R. China

**Keywords:** magnesium-based metal, mineralized collagen, Wnt signaling pathway, tumor microenvironment

## Abstract

One of the most prevalent malignant tumors in women is cervical cancer. Conventional chemoradiotherapy was frequently limited by significant side effects and acquired drug resistance. Consequently, there is an urgent need for high-performance biomaterials that effectively suppress tumor growth while exhibiting minimal off-target toxicity. Magnesium alloys represented a promising platform for anti-tumor applications due to their bioactive degradation products. This study developed novel magnesium alloy-mineralized collagen composite scaffolds and systematically evaluated their surface properties. Comprehensive *in vitro* and *in vivo* experimental models were used to elucidate the scaffolds’ anti-tumor mechanisms. The results of this study demonstrated that magnesium alloy-mineralized collagen composite scaffolds significantly inhibit tumor cell invasion and metastasis while promoting cancer cell death. Based on *in vivo* and *in vitro* studies, this study showed that the degradation products of magnesium alloy-mineralized collagen composite scaffolds target epithelial–mesenchymal transition through the Wnt/β-catenin/TCF7 signaling pathway. These findings established a robust experimental foundation for advancing magnesium alloy-mineralized collagen composite scaffolds as next-generation biodegradable adjunctive therapeutic materials for cervical cancer treatment. The synergistic combination of biocompatibility and tumor-targeted activity positions this material as an innovative platform for circumventing shortcomings in existing clinical regimens.

## Graphical abstract

**Figure:**
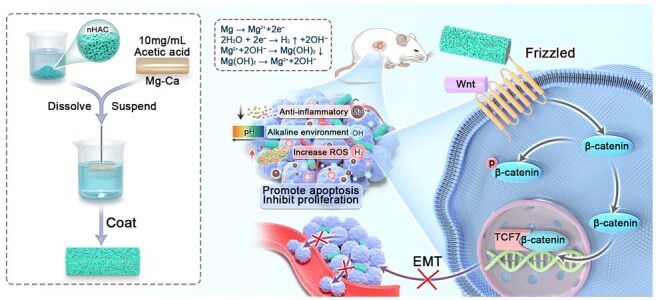

## Introduction

According to the database of 2020 Global Cancer Epidemiology (GLOBOCAN), cervical cancer accounts for approximately 604 000 newly diagnosed cases and results in around 342 000 fatalities worldwide [1]. Xinjiang is one of the high-incidence areas of cervical cancer in China, and cervical cancer is closely related to HPV infection, with common genotypes of HPV16, HPV33 and HPV52 [2]. The most common causes of death from cervical cancer are invasion and distant metastasis. The mechanisms of its occurrence are not yet fully understood. Most of early-stage patients have no obvious clinical manifestations, and in the middle and late stages, the invasion of tumor to the surrounding tissues or organs often poses a serious threat to the patients’ lives. Currently, radical surgery is often used for early-stage cervical cancer patients, but radiation or chemotherapy is needed for patients with large tumors and inflammation and metastasis in advanced stages. Cisplatin and paclitaxel are widely utilized chemotherapy agents for cervical cancer treatment. However, their clinical application is often hindered by the emergence of drug resistance and a range of severe complications, including allergic manifestations and impaired bone marrow function, hearing loss, kidney toxicity and digestive system complications. These challenges have significantly restricted their effectiveness and contributed to disease progression in certain individuals [3]. Therefore, the research of improving drug targeting, reducing tumor resistance and toxic side effects has been the difficulty and hotspot of anti-tumor drug research. Currently, biomaterials therapy has a great application prospect in tumor adjuvant therapy, based on the fact that biomaterials have the following roles: (1) better control of the therapeutic range and intensity of treatment, effectively reducing adverse reactions and improving therapeutic efficiency on the basis of chemotherapy, radiotherapy, thermotherapy and reactive oxygen species (ROS) dynamic therapy. (2) maximizing the effect of tumor killing and reducing dose-dependent adverse reactions as a new type of targeted drug delivery system and specific treatment. (3) iInhibiting tumor cell invasion and metastasis, and reducing tumor recurrence.

In recent years, degradable magnesium (Mg) alloys are increasingly being explored as promising candidates for orthopedic implants due to their advantageous properties, and Mg degradation has a cytotoxic effect on tumor cells with potential anti-tumor activity [4, 5]. In addition, localized implantation offers a way to circumvent the systemic adverse effects associated with therapeutic drugs, enabling targeted delivery of implantable H_2_ therapy. Some research has indicated that Mg and its alloys may exhibit potential anti-cancer properties via several mechanisms: the degradation of Mg and its alloys releases byproducts such as Mg^2+^, OH^−^ and H_2_, which collectively contribute to anti-tumor effects. These components inhibit tumor cell proliferation by promoting the accumulation of free radicals. Specifically, Mg^2+^ and H_2_ have been shown to arrest tumor cells in the G0/G1 phase of the cell cycle [6, 7], while the release of OH^−^ elevates the extracellular pH around tumor cells, creating an unfavorable environment for their growth [8]. However, the most significant obstacle to the clinical application of Mg-based metals is their rapid and uncontrollable corrosion or degradation behavior in chloride-containing (e.g. body fluids). In order to make Mg-based metal materials last in the complex environment *in vivo*, the preparation of new Mg-based composites by adjusting the materials composition and organizational structure, adopting new material preparation techniques and surface treatment coatings to reduce the corrosion rate of the materials and improve their biocompatibility is a highly discussed subject within the realm of biomaterials science [9].

Mineralized collagen (nHAC) consists mainly of collagen type I and nanohydroxyapatite [10]. Nanohydroxyapatite has the advantages of high biocompatibility and low cytotoxicity, which can kill tumor cells by changing their ultrastructure and function [11]. At the same time, it can mineralize the local collagen fibers of tumor tissues to prevent tumor metastasis and invasion. Therefore, it shows good potential for application in killing tumor cells and preventing tumor metastasis [12]. Nanohydroxyapatite has been shown to inhibit the tumor, such as osteosarcoma, gastric, colon and breast cancers [13]. It has been reported that nano hydroxyapatite can enter the cytoplasm through the cell membrane via the endocytosis pathway, ultimately inhibiting the growth of osteosarcoma cells [14]. Nanohydroxyapatite can also induce apoptosis by disrupting mitochondrial membrane sites [15]. nHAC consists mainly of collagen type I and nanohydroxyapatite. There is more information on the use of nHAC in areas such as orthopedics, but there is little literature on its use in the oncology field. Therefore, we speculate whether nHAC also has some anti-tumor effects.

The Wnt/β-catenin signaling pathway is a classical type of Wnt pathway with functions such as regulating cell proliferation, migration and differentiation [16]. Research has demonstrated that the activation of the Wnt/β-catenin signaling pathway can promote the proliferation, migration and invasive behavior of cancer cells [17]. Increased expression of β-catenin, increased nuclear entry and binding to LEF/TCF are followed by the activation of its downstream genes involved in tumor proliferation and invasion [18]. Additionally, the Wnt/β-catenin signaling pathway serves as an upstream regulator of EMT. Its activation frequently triggers EMT in various cancer cells, enhancing their ability to proliferate and metastasize [19]. During tumor progression, a variety of tumor cells show changes in plasticity through morphological and phenotypic transformations [20, 21]. EMT is characterized by the reduced expression of the epithelial marker E-cadherin, along with the increased expression of mesenchymal markers such as Vimentin and N-cadherin [22]. However, Wnt pathway inhibitors can also have adverse effects on their targets. Although the Wnt signaling pathway plays a key role in carcinogenesis and tumor development, there are no approved drugs targeting this pathway. Inhibition of the Wnt signaling pathway via other bypass or upstream regulatory pathways, resulting in fewer side effects [23, 24].

At present, it is not clear whether Mg alloys and their degradation products can inhibit EMT through Wnt signaling pathway to achieve anti-tumor effect in cervical cancer, and further studies are needed. Therefore, the preparation of optimized Mg-based metal composites and the study of their anti-tumor effects and mechanisms *in vitro* and *in vivo* will provide a new strategy and experimental basis for the development of novel biofunctional adjuvant therapeutic materials for cervical cancer. Firstly, the Mg alloys were subjected to freeze-drying in an nHAC solution. Mg alloys-nHAC composite scaffolds were co-cultured with cervical cancer SiHa, HeLa cells. *In vitro*, cell metabolism, apoptosis, and cell scratching, cell cloning, etc. analyses were detected to observe whether Mg-Ca-nHAC composite scaffolds could inhibit the invasion and metastasis of cervical cancer cell lines (SiHa, HeLa cells). Subsequently, this study was further validated by implanting the composite scaffolds into a nude mouse model conducting *in vivo* animal experiments. This investigation shed light on the therapeutic potential and underlying mechanisms of Mg-Ca-nHAC in cervical cancer treatment, proposing an innovative biomaterial-based approach to enhance adjuvant therapies for cervical cancer.

## Materials and methods

### Materials preparation

The Mg-Ca alloys specimen, fabricated as a rod with a diameter of 10 mm and a thickness of 10 cm, contained 1 wt% calcium and exhibited a mass of 12.45 g. This material was procured from a higher education institution in Beijing Union University. The nHAC bone powder was supplied by Allgens Medical Technology Co., Ltd. Its nano-based apatite accounts for 45% ± 5%. Its porosity was 80–90%, and pore size was 50–500 nm. As biomaterial composites, its relevant performance evaluation can be conducted in accordance with the GB/T 16886 series of standards for biological evaluation. Mg-Ca alloys:nHAC bone powder ratio (0.1 g/mL:60 mg/mL). During the preparation of Mg-Ca-nHAC, nHAC (10 mg/mL acetic acid solution) was uniformly dispersed onto the surface of the Mg-Ca alloys via suspension method.

### Analysis of composite scaffolds properties

#### Surface structure characteristics

Take trace samples and directly stick them to the conductive adhesive, and use the Oxford Quorum SC7620 sputtering coating instrument to spray gold for 45 s (the specific spraying time depends on the sample/test requirements), with a spraying gold of 10 mA. Subsequently, conduct morphological and energy spectrum analyses in a dry environment. Position the ATR accessory within the spectrometer’s optical path and perform an air background scan. Ensure the block or thin film sample is firmly pressed against the ATR crystal surface. Acquire the sample’s infrared spectrum with a resolution of 4 cm^−1^ and 32 scans. For atomic force microscopy (AFM), secure the solid or thin film sample onto a glass slide and analyze it using the Bruker Dimension Icon AFM system.

#### Materials degradation detection

The concentrations of Mg^2+^ and Ca^2+^ were measured using (ICP-MS) Agilent 5110 OES, USA. To evaluate *in vitro* degradation, the materials were immersed in simulated body fluid, a solution with ion concentrations comparable to human plasma. All degradation experiments were conducted at a constant temperature of 37°C in a simulated physiological environment. The experiments were conducted under controlled conditions, with the pH adjusted to 4.5, 5.5 and 7.4. The samples were agitated at a constant speed of 60 rpm. The primary degradation byproducts of the Mg-Ca-nHAC composites were identified as Mg^2+^ and Ca^2+^.

#### Measurement of H_2_ generation

Composite scaffolds were immersed in centrifuge tubes for static observation. The composite scaffolds were subjected to H_2_ measurements in PBS and the amount of H_2_ was measured at different pH values and different time periods. Additionally, the H_2_ released by the composite scaffolds in PBS was quantitatively analyzed using gas chromatography (Agilent’s three-valve, four-column-FID+TCD dual detector).

#### XRD experimental testing

Fill the sample holder uniformly with the prepared Mg-Ca-nHAC composite scaffolds samples and press them flat to form a smooth surface. Using the Bruker D8 ADVANCE with a 90-position autosampler. Conducted *in situ* testing at 10°–90°, 6°/min. Switch on the diffractometer and cooling system, then initialize the goniometer. Securely mount the sample holder onto the sample stage, close and lock the protective door and verify that the safety interlock is engaged. Configure the scanning range within the control software. After confirming all safety precautions are in place, initiate the measurement program. The instrument will automatically complete the scan and save the data file.

#### Porosity testing

Operational Reference Standard: GB/T 21650.1-2008. The mercury intrusion porosimetry method is based on the principle that non-wetting liquids can be forced into the pores of porous materials under pressure. According to the Washburn equation, the applied pressure (P) correlates directly with the radius r of the pores through which mercury can penetrate. By incrementally increasing the pressure and measuring the volume of mercury entering the pores, information such as the material’s pore size distribution, total pore volume and porosity can be obtained.

### *In vitro* investigation

#### Cell culture and experimental groups

HeLa/SiHa/H8 cells were purchased from Shanghai Saibai Kang Biotechnology Co., Ltd. Complete medium was prepared by mixing MEM medium and FBS at a ratio of 9:1. Cells were cultured in a 5% CO_2_, 37°C incubator. Firstly, the research experiment groups were divided as follows: control, Mg-Ca, nHAC, Mg-Ca-nHAC. Secondly, LGK974 (NVP-LGK974, WNT974) is an effective specific P inhibitor that inhibits the Wnt signaling pathway. Take HeLa and SiHa cells in the logarithmic growth phase, adjust the cell concentration, inoculate them into appropriate wells and divide them into groups according to control, inhibitor LGK974, Mg-Ca-nHAC, and inhibitor LGK974+Mg-Ca-nHAC.

#### CCK8 detection

HeLa/SiHa/H8 cells were harvested, and cell concentrations were adjusted, seeded into 96-well plates at 8 × 10^3^/well, treated by groups as described above, and cultured for 24 h/48 h/72 h in a 5% CO_2_, 37°C incubator. Subsequently, 100 μl of fresh medium containing 10% CCK-8 reagent was added to each well. The plates were then incubated at 37°C in a 5% CO_2_ atmosphere for 2 h. Following incubation, the absorbance at 450 nm was quantified. The experiment included groups: control, Mg-Ca, nHAC, Mg-Ca-nHAC. LGK974 (NVP-LGK974, WNT974) is a specific Wnt pathway inhibitor. Log-phase HeLa and SiHa cells were adjusted for concentration, seeded into wells and grouped as control, LGK974, Mg-Ca-nHAC and LGK974+Mg-Ca-nHAC.

#### Rhodamine ghost pen cyclic peptide stain

HeLa/SiHa cells were harvested, and cell concentrations were adjusted, seeded into laser confocal dishes at 6 × 10^4^ cells/well and incubated overnight in a 5% CO_2_, 37°C incubator. As grouped as above, cultures were grown in a 5% CO_2_, 37°C thermotemperature incubator for 24 h. Serum-free medium was diluted in rhodamine-labeled medium to 1/200, added to DAPI and adjusted to 5 μg/ml. PBS was washed twice to remove excess serum. The cells were fixed in 1000 μl/well with 4% formaldehyde solution at room temperature for 10 min. PBS was washed twice, 1000 μl/well of rhodamine-labeled ghost pen cyclic peptide and DAPI working solution and Samples were maintained at ambient temperature for half an hour. Marked with rhodamine with a 559 nm laser and DAPI with a 405 nm laser.

#### Mitochondrial membrane potential

HeLa and SiHa cells were inoculated into 6-well plates according to 3 × 10^5^ cells/well, 5% CO_2_, 37°C constant temperature incubator until the cells adhered to the wall. The experimental group was added with the original solution of the extract. Prepare the samples according to the instructions of the kit, and then detect them on the flow cytometer.

#### Live/dead staining

HeLa and SiHa cells concentration adjusted, and seeded into 24-well plates at 5 × 10^4^/well. In the following, cells were grown in a 5% CO_2_, 37°C incubator for 24 h. A 1:10 dilution of Calcein-AM and PI was prepared in the dye dilution buffer. Cells in 24-well plates were washed once with PBS to remove excess serum. Each well received 1 ml of the solution, followed by a 15-min incubation period at 37°C.

#### Cell cyle detection

HeLa and SiHa cells harvested at the mid-logarithmic phase were quantified and their densities adjusted to the desired concentration and seeded into 6-well culture plates at 3 × 10^5^/well and grown overnight in a 5% CO_2_, 37°C incubator to make cells adherent. Groups were treated as described above and cultured for 24 h. Cells were collected by pancreatic enzyme digestion, after one wash with PBS, Add 500 μL/tube to precold ethanol suspended cells, and the cells were fixed at −20°C. Add 20 μl/tube of RNase A, incubation in 37°C for 30 min. Add 400 μl/tube of PI dye solution. Cell cycle changes were measured by flow cytometric RED-B channels.

#### Cell apoptosis detection

HeLa and SiHa cells harvested at the mid-logarithmic phase were quantified and their densities adjusted to the desired concentration, the cells were inoculated into 6-well plates according to 3 × 10^5^ cells/well. The experimental group was added with the original solution of the extract. Following guidelines provided in the kit manual, the staining process was executed. Flow cytometry was performed on the machine.

#### ROS detection

HeLa and SiHa cells harvested at the mid-logarithmic phase were quantified and their densities adjusted to the desired concentration and seeded into 24-well plates at 5 × 10^4^/dish overnight in a 5% CO_2_, 37°C incubator. Groups were treated as described above and cultured for 24 h. Then added 1 mL reactive oxygen-positive control working solution, and incubated for 30 min. After the incubation, medium was discarded from all experimental groups except blank, washed with PBS 3 times, added 1 ml DCFH-DA working solution (10 μM) and incubated for 20 min.

#### Cell migration

HeLa and SiHa cells at the mid-logarithmic phase were harvested, quantified and adjusted to the desired concentration. Subsequently, the cells were diluted with 2% serum medium and seeded into 24-well plates at a density of 6 × 10^4^ cells/well. The experimental group was added to the original solution of the extracts. The cells were wiped off from the upper compartment by a swab, the cells stained with crystal violet were photographed by a microscope, and the migration of the cells was recorded statistically. Experimental groups: control, Mg-Ca, nHAC, Mg-Ca-nHAC. LGK974 (NVP-LGK974, WNT974) is a specific Wnt pathway inhibitor. Log-phase HeLa and SiHa cells were adjusted for concentration, seeded into wells and grouped as control, LGK974, Mg-Ca-nHAC, LGK974+Mg-Ca-nHAC.

#### Cell invasion

HeLa and SiHa cells harvested at the mid-logarithmic phase were quantified, their densities adjusted to the desired concentration and then diluted with 2% serum medium. These cells were inoculated into 24-well chambers (8 × 10^4^ cells/well) that had been pre-lined with diluted matrix gel. The experimental groups were added with the extracted liquid stock solution outside the chambers. Cells labeled with crystal violet were imaged using microscopy, and the invasive capacity was quantified statistically.

#### Scratch detection

HeLa and SiHa cells harvested at the mid-logarithmic phase were quantified, and their densities were adjusted to the desired concentration. The cells were then diluted with 5% serum medium and inoculated into 12-well plates at 2 × 10^5^ cells/well. They were incubated in a 37°C constant temperature incubator with 5% CO_2_. Once the cells had attached to the wall, a 200 μl yellow pipette tip was used to scratch the cells vertically in the wells. The cells were washed twice with PBS to remove floating cells from the scratches, and the serum concentration in the medium of each group was halved. The scratched area was photographed and the changes of the scratched area were counted at 0, 24, 48 and 72 h, respectively, and Image J was used for the statistic.

#### Plate clone experiment

HeLa and SiHa cells harvested at the mid-logarithmic phase were quantified, their densities adjusted to the desired concentration, and then inoculated into 6-well plates at 500 cells/well. They were cultured in a 37°C incubator with 5% CO_2_ for 14 days, with the medium changed every 3 days. Add 4% paraformaldehyde and fix for 30 min, discard paraformaldehyde, wash with PBS 3 times and add crystal violet staining for 2 h. Discard the staining solution, wash with PBS 3 times and take pictures with gel imager.

#### Transcriptomics assay

HeLa and SiHa cells harvested at the mid-logarithmic phase were quantified and inoculated into 10 cm dishes at 1 × 10^6^ cells/dish. They were cultured in a 37°C constant temperature incubator with 5% CO_2_ until adhered to the wall. The experimental group was added to the original extract. After being cultured in a 37°C constant temperature incubator for 24 h, respectively. The cells were collected by trypsin digestion. Then, 1 ml of Trizol was added to each dish, and the cells were thoroughly dissociated by pipetting. The resulting lysate was rapidly frozen in liquid nitrogen and subsequently sent for transcriptome analysis on dry ice.

#### Western blot assay

The experiment included groups: control, Mg-Ca, nHAC, Mg-Ca-nHAC. Additionally, LGK974 (NVP-LGK974, WNT974), a specific Wnt pathway inhibitor, was used. Log-phase HeLa and SiHa cells were adjusted for concentration, seeded into appropriate wells and further grouped as control, LGK974, Mg-Ca-nHAC and LGK974+Mg-Ca-nHAC.The experimental group was treated with the original solution of the extract and incubated in a 37°C, 5% CO₂ incubator for 24 h. Following incubation, the supernatant was discarded, and the cells were washed once with PBS. The collected supernatant was then subjected to WB detection.

#### RT-PCR assay

The experiment included groups: control, Mg-Ca, nHAC, Mg-Ca-nHAC. LGK974 (NVP-LGK974, WNT974), a specific Wnt pathway inhibitor, was used in additional groups: control, LGK974, Mg-Ca-nHAC and LGK974+Mg-Ca-nHAC. Log-phase HeLa and SiHa cells were adjusted for concentration, seeded into appropriate wells and treated for 24 h before undergoing RNA extraction, reverse transcription and PCR detection as per standard protocols. PCR primer design was shown in Table 1.

**Table 1.** Oligonucleotide primer pairs designed for reverse transcription PCR

Target	Primer sequence
Wnt1-F	CGATGGTGGGGTATTGTGAAC
Wnt1-R	CCGGATTTTGGCGTATCAGAC
β-catenin-F	AAAGCGGCTGTTAGTCACTGG
β-catenin-R	CGAGTCATTGCATACTGTCCAT
TCF7-F	CTGGCTTCTACTCCCTGACCT
TCF7-R	ACCAGAACCTAGCATCAAGGA
E-cadherin-F	CGAGAGCTACACGTTCACGG
E-cadherin-R	GGGTGTCGAGGGAAAAATAGG
N-cadherin-F	TCAGGCGTCTGTAGAGGCTT
N-cadherin-R	ATGCACATCCTTCGATAAGACTG
Vimentin-F	GACGCCATCAACACCGAGTT
Vimentin-R	CTTTGTCGTTGGTTAGCTGGT
Snail-F	AGAGCTGACCTCCCTGTCA
Snail-R	TGAAGTAGAGGAGAGAGAGAGAGAGAGAGAGA
Twist-F	GTCC GCAGTCTTACGAGGAG
Twist-R	CCAGCTTGAGGGTCT GAATC
β-actin-F	TGACGTGGACATCCGCAAAG
β-actin-R	CTGGAAGGTGGACAGCGAGG
GAPDH-F	GGAGCGAGATCCCTCCAAAAT
GAPDH-R	GGCTGTTGTCATACTTCTCATGG

PCR primer design for Wnt1, β-catenin, TCF7, E-cadherin, N-cadherin, vimentin, Snail, Twist, β-actin, and GAPDH.

### *In vivo* investigation

#### Animals model construction

The research experiment groups were divided as follows: control, Mg-Ca, nHAC, Mg-Ca-nHAC. After the animals were acclimatized and fed for 1 week, 100 µl of HeLa-Luc cells at a concentration of 2 × 10^7^ cells/ml were subcutaneously injected into each of the 12 animals. Tumor growth was monitored and measured every 3/4 days until the tumor diameter reached 80–100 mm³. Furthermore, this study established the model based on four groups: control group, inhibitor LGK974 group, Mg-Ca-nHAC group and inhibitor LGK974+Mg-Ca-nHAC group. Cell digestion was adjusted to a concentration of 2 × 10^7^ HeLa cells/ml, with 2 × 10^6^ HeLa cells injected subcutaneously per animal.

#### Experimental grouping and treatment

Control group was the model group without intervention. For the nHAC group, 6 mg nHAC solution was injected into the tumor *in situ* with a needle. For the Mg-Ca treated group, the Mg-Ca alloy rod size was 1 × 7 mm. In the Mg-Ca-nHAC alloy group, the Mg-Ca alloy rods (1 × 7 mm) were first treated with a coating of 6 mg of nHAC solution and then a sterile 5 ml disposable needle was used to make a small hole in the side of the tumor before implanting the materials (without penetration), and the materials were gently rotated into the tumor with tweezers until they were completely entered. Three animals were selected from each group. Tumor sizes were measured and mice were weighed twice a week, and tumor volume was calculated as follows: tumor volume=(*L*×*W*^2^)/2, where *L* is the length of the tumor and *W* is the width of the tumor. *In vivo* imaging was performed at 0, 7 and 14 days after treatment, and 3 animals were taken from each group. Sodium D-fluorescein at a concentration of 15 mg/ml was injected at 100 μl/10 g body weight 10 min before imaging. The mice were euthanized after the experimental endpoint, and the tumor with surrounding skin, heart, liver, spleen, lung and kidney was fixed with 4% (v/v) paraformaldehyde and embedded in paraffin, and then stained with HE for histopathological analysis. At 14 days, all tumor were taken. The tumor tissues were subjected to HE, TUNEL and Ki67 staining to observe tumor cell apoptosis. Immunohistochemical staining was performed for Wnt1, β-catenin and TCF7. Additionally, PCR was used to detect the expression levels of E-cadherin, N-cadherin and Vimentin genes. The reverse validation experiment was conducted as follows: control group was the model group without intervention. For the LGK974 inhibitor, prepare a 5 mg/kg solution in PBS. For the Mg-Ca-nHAC, the treatment method was as above. Tumor size was measured and mouse body weight recorded on Days 0, 3, 6, 9, 12 and 14 post-treatment. Tumor volume was calculated as follows: tumor volume=(*L*×*W*^2^)/2, where *L* is the length of the tumor and *W* is the width of the tumor. At 14 days, all tumors were taken. The tumor tissues were subjected to HE, Tunel and Ki67 staining to observe tumor cell apoptosis. Immunohistochemical staining for Wnt1, β-catenin, TCF7, E-cadherin, N-cadherin, Vimentin after sampling. All animal experiments were conducted in accordance with ethical guidelines and were approved by the Institutional Ethics Committee of Xinjiang Medical University (Approval No. IACUC-20220725-24).

### Statistical analysis

For all experiments, at least three samples in one group were needed to be analyzed. One-way analysis of variance (ANOVA) was used for the analysis of differences between the two groups. It was determined whether there was statistical significance between the groups of samples. The mean ± standard deviation of each group was used for quantitative data. All differences between groups were considered statistically significant when *P* < 0.05.

## Results

### Morphology and characterization

**Figure 1.**
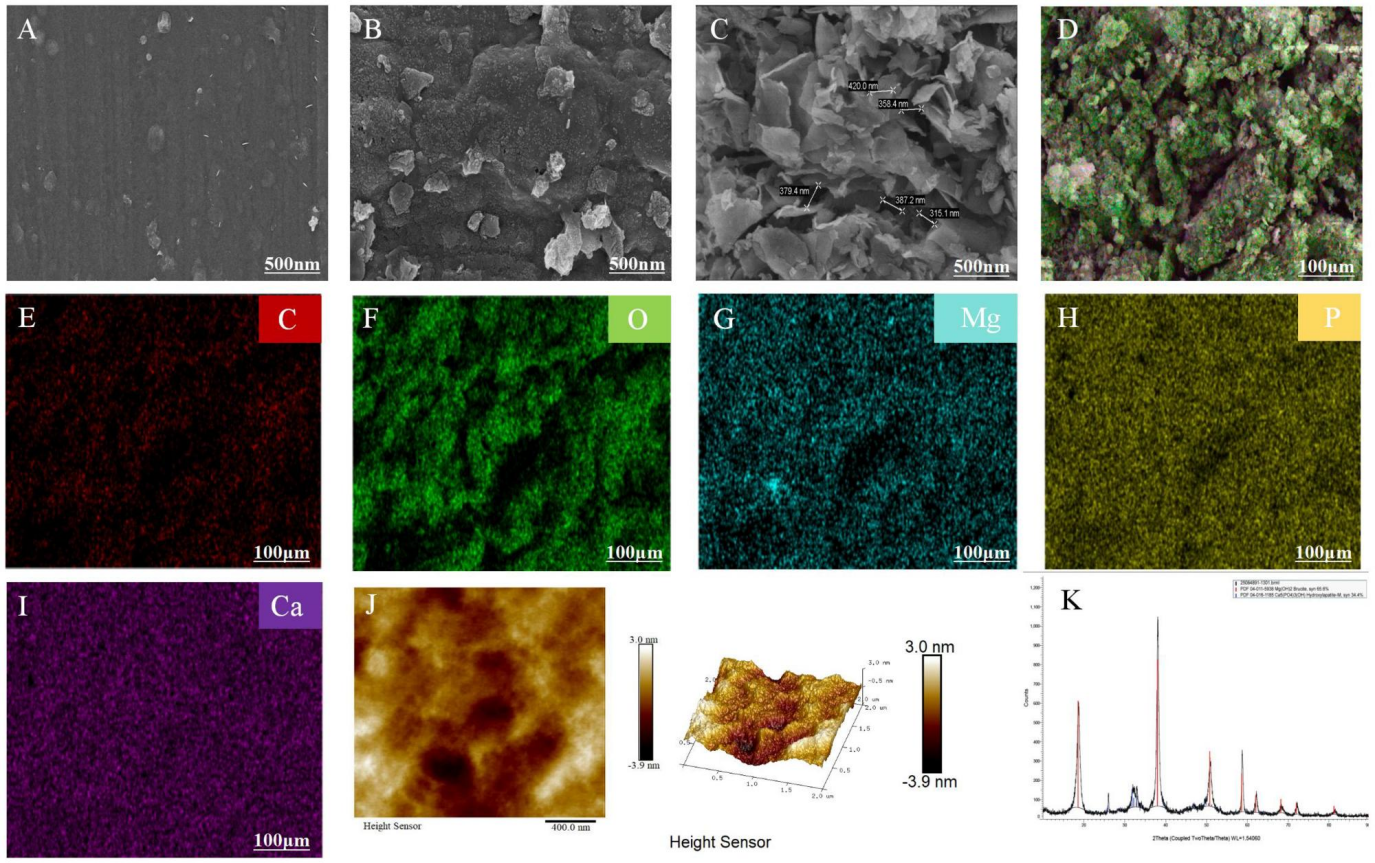
Preparation and characterization of Mg-Ca-nHAC composite scaffolds. (**A–C**) The representative SEM images of Mg-Ca scaffolds, nHAC scaffolds, Mg-Ca-nHAC composite scaffolds. (**D–I**) The representative EDS element mapping of Mg-Ca-nHAC composite scaffolds. (**J**) AFM test to check the surface flatness of the Mg-Ca-nHAC composite scaffolds. (**K**) XRD diffractogram of Mg-Ca-nHAC composite scaffolds (04-011-5938 Mg(OH)_2_, 04-016-1185 hydroxyapatite).

Figure 1A–C showed the experiments of scanning electron microscope (SEM), the results were shown in Figure 1A for Mg-Ca group, Figure 1B for nHAC group and Figure 1C for Mg-Ca-nHAC group. As shown in the Mg-Ca group, there had a little oxidation manifestation on the surface, Mg metal is an active metal, which could be oxidized quickly in the air. This study employed nHAC coatings to mitigate accelerated oxidation, as presented in Figure 1B. nHAC was a composition of type I collagen and nanohydroxyapatite, and has a porous structure. As shown in Figure 1C, it can be seen that there are multiple pore sizes, the pore size range was in the nanometer scale. Therefore, detecting the composition of composite materials by energy spectroscopy, as shown in Figure 1D–I, it can be found that the composites contain a large amount of carbon (C), oxygen (O), phosphorus (P), calcium (Ca) and other elements. In addition, we did AFM test to check the surface flatness of the composites, in which results showed that the surface of the composite is basically flat and uniform coating treatment (Figure 1J). Furthermore, as depicted in Figure 1K, XRD analysis was conducted on the Mg-Ca-nHAC composite scaffolds in this study. The spectrum revealed that the inorganic crystalline components of the Mg-Ca-nHAC composite scaffolds material comprise Mg(OH)_2_ (65.6%) and hydroxyapatite (34.4%) structures.

### Materials degradation detections and H_2_ release

**Figure 2.**
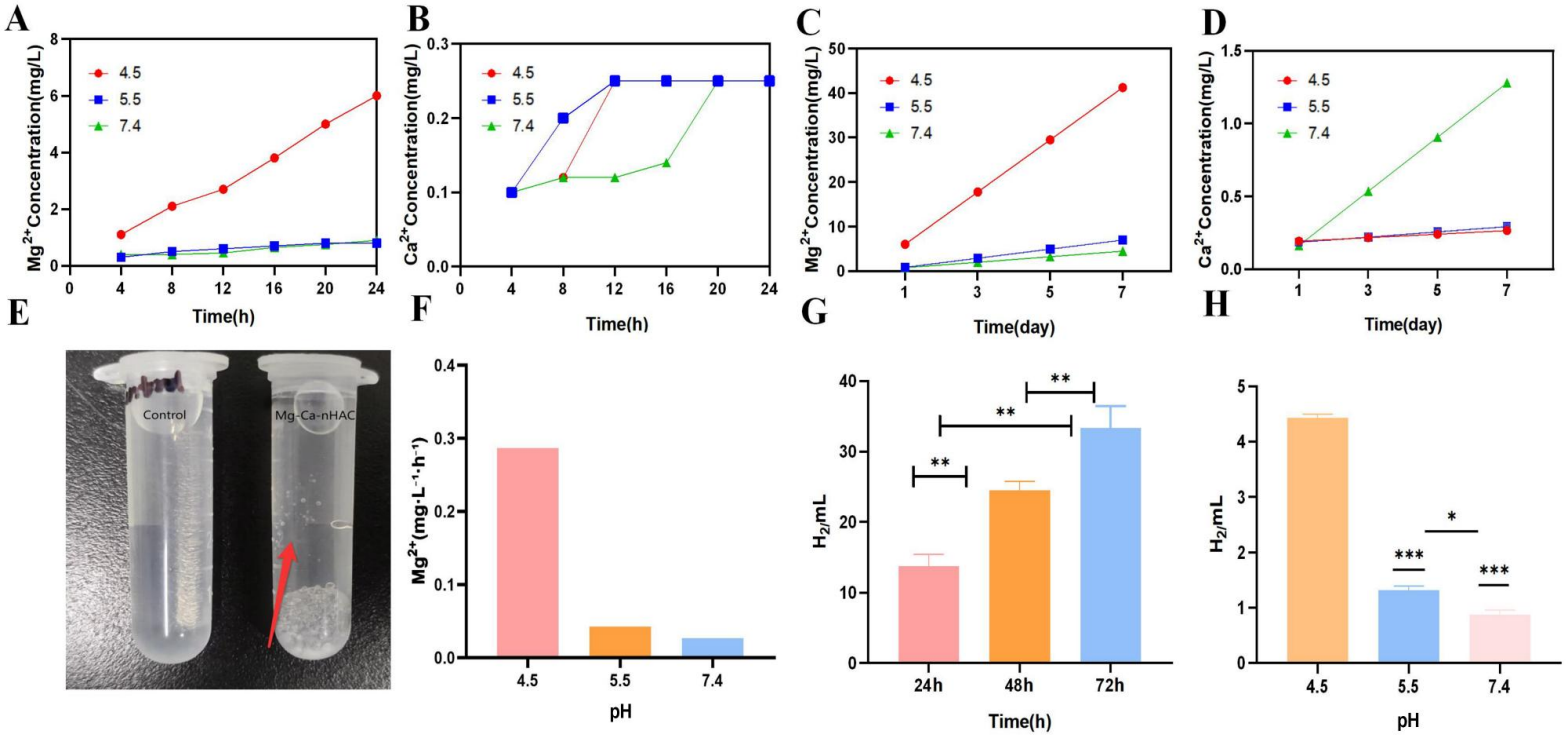
Material properties testing. (**A**) Detection of Mg^2+^ concentration changes in Mg-Ca-nHAC materials using ICP-MS method at different PH. (**B**) Detection of Ca^2+^ concentration changes in Mg-Ca-nHAC materials using ICP-MS method at different PH. (**C**) Determination of Mg^2+^ concentration changes in Mg-Ca-nHAC materials at 1, 3, 5 and 7 days under varying pH conditions using the ICP-MS method. (**D**) Determination of Ca^2+^ concentration changes in Mg-Ca-nHAC materials at 1, 3, 5 and 7 days under varying pH conditions using the ICP-MS method. (**E**) Photograph indicating H_2_ generation in PBS from Mg-Ca-nHAC composite scaffolds. (**F**) Mg^2+^ release rate under different pH conditions. (**G**) H_2_ gas chromatography experiments in 24 h, 48 h, 72 h (**P < 0.01). (**H**) H_2_ generation of Mg-Ca-nHAC composite scaffolds in PBS solutions with different pH values measured by gas chromatography in 72 h (***P < 0.001 vs pH 4.5, *P < 0.05).

In order to better simulate the changes of degradation products of composite scaffolds in the tumor microenvironment, this study employed ICP-MS to analyze the release of Mg^2+^ and Ca^2+^ under varying pH conditions. According to Figure 2A–D, the acidic environment was more favorable to promote the release of Mg^2+^. Research has demonstrated that the tumor microenvironment exhibits a weakly acidic [25]. According to the above results, we suspected whether Mg-based composite scaffolds can exert anti-tumor effects by degrading products Mg^2+^ and H_2_. According to Figure 2E, it was observed that the composite material immersed in PBS released a large amount of gas. Based on the chemical reaction, it was speculated that the gas could be hydrogen. To further detect the composition of the degradation products, gas chromatography experiments were conducted, which showed that the hydrogen content increased significantly with the immersion time in the composite material (Figure 2G). Figure 2F result indicated that the degradation rate of Mg^2+^ was also influenced by pH, with a decrease in pH accelerating the degradation rate. Moreover, the release of hydrogen varied with the change of pH, and the lower pH was more favorable to promote the release of hydrogen (Figure 2H). The porosity of Mg-Ca-nHAC composite scaffolds was determined using mercury porosimetry. As shown in Supplementary Information Figure S1, the pore volume distribution predominantly ranged between 0 and 100 nm. Instrumental analysis revealed a porosity of 4.5081%.

### *In vitro* experiment

#### In vitro biocompatibility test

**Figure 3.**
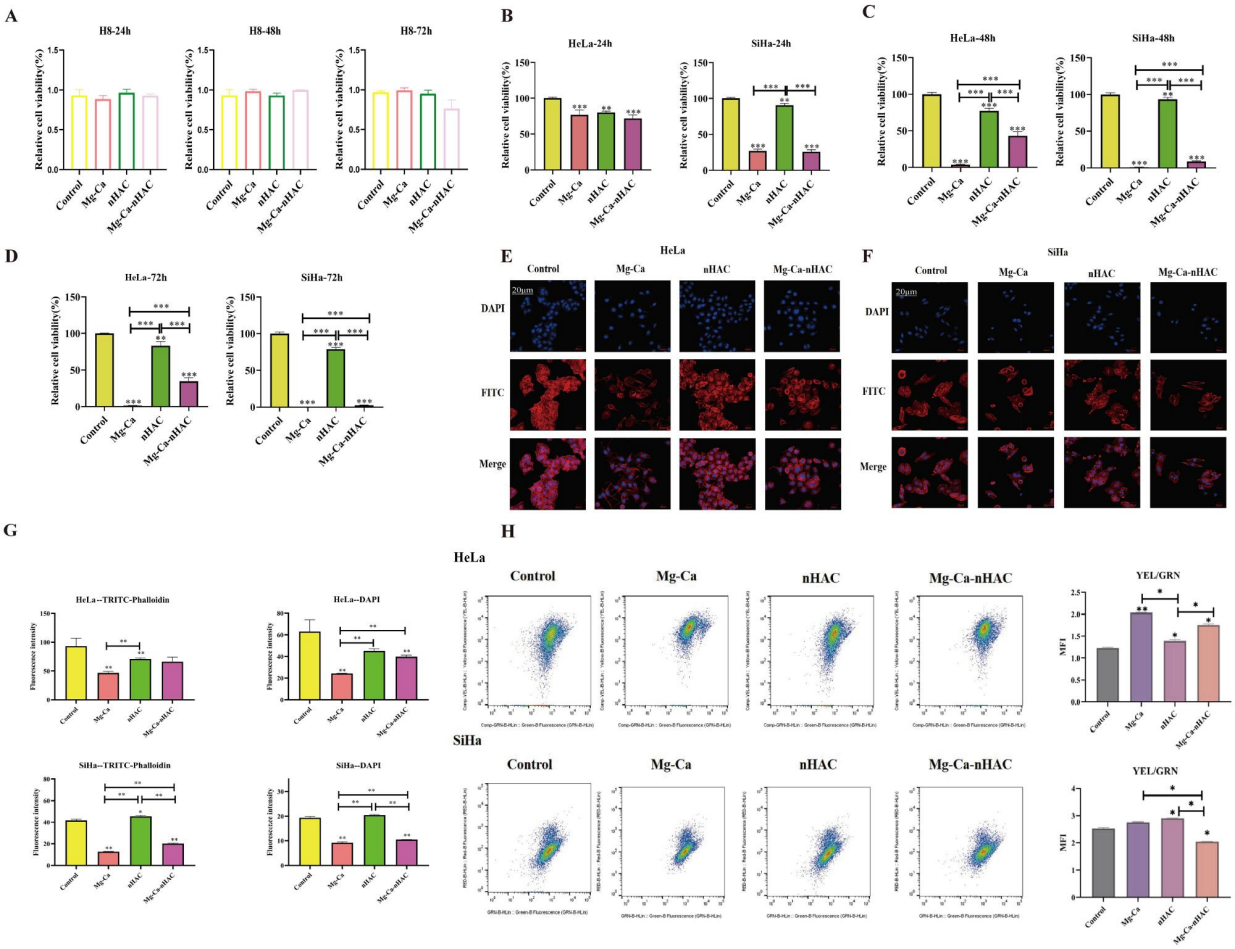
*In vitro* anti-tumor assays. (**A–D**) In different cell lines (H8/HeLa/SiHa), the cell proliferation activity of different treatment groups was detected at 24, 48 and 72 h through the CCK8 experiment (**P < 0.01,***P < 0.001). (**E–G**) Detection of cytoskeletal extension in different treatment groups at 24 h by staining with ghost closed loop peptide and the fluorescence intensity was quantified (scale bar: 20 μm, **P < 0.01). (**H**) Mitochondrial membrane potential was detected and quantitatively analyzed (*P < 0.05,**P < 0.01).

Firstly, in order to assess the safety of the biological materials used in this study, the CCK8 assay was performed on normal cervical epithelial cell lines (H8) (Figure 3A). The results revealed that there were no significant differences between the treatment groups and the control groups. In order to detect the proliferative effect of each group of materials on cervical cancer cells, the study used the CCK8 detection assay and the absorbance at 450 nm of the assay was measured after 24 h, 48 h, 72 h of incubation, respectively. As shown in Figure 3B–D, at 24 h, for HeLa cells, each group of experimental materials had a significant inhibitory effect on the proliferation of HeLa cells compared with the blank control group. For SiHa cells, the treatment groups had a significant inhibitory effect, especially the two groups of Mg-Ca and Mg-Ca-nHAC composite scaffolds had the most significant inhibitory effect. At 48 and 72 h, for HeLa and SiHa cells, the treatment group had a significant inhibitory effect, especially the Mg-Ca and Mg-Ca-nHAC composite scaffolds groups had the most obvious inhibitory effect. SiHa cells were the most sensitive to the action of the treatment group, and the inhibition was better than that of HeLa cells. Especially with the increase of time, the toxic effect of Mg-Ca alloys increased, the inhibitory effect of nHAC on the cells was weaker and the toxic effect of Mg-Ca-nHAC composite scaffolds on the cells was stronger. In addition, in this study, rhodamine-labeled ghost pen cyclic peptide was used to detect HeLa and SiHa cytoskeletons, and the experimental results were shown in Figure 3E–G: in SiHa cells, the cytoskeleton stretching was not obvious in the special Mg-Ca and Mg-Ca-nHAC composite scaffolds groups, the cell adhesion was relatively poor and there was a significant difference compared with the control group, which was statistically significant. Based on the chemical reaction, the gas was speculated to be hydrogen. To further identify the degradation products, gas chromatography experiments were conducted, showing that hydrogen content increased significantly with immersion time in the composite material. It has been reported that high concentration of H_2_ kills cancer cells by inhibiting cellular mitochondrial respiration [26]. Therefore, we evaluated the inhibitory effect of Mg-Ca-nHAC composite scaffolds on mitochondrial respiration by determining the change in membrane potential using JC-1 dye as a fluorescent indicator. In Figure 3H, JC-1 dye was used as a fluorescence indicator to evaluate the effect of different treatment groups on inhibiting mitochondrial respiration. The decrease of cell membrane potential could be detected through the transformation of JC-1 from yellow fluorescence to green fluorescence, and the transformation of JC-1 fluorescence color could be used as an early detection index of apoptosis [27, 28]. The experimental results showed that for HeLa cells, the treatment group had no obvious inhibitory effect on mitochondrial membrane potential, while for SiHa cells, especially for Mg-Ca-nHAC composite scaffolds groups, the inhibitory effect was more obvious, indicating that mitochondrial function was seriously damaged. It may be that the degradation product H_2_ causes mitochondrial dysfunction, further promoting the death of cancer cells. ROS detection showed the cells were in oxidative stress, and reduced ROS levels promoted increased mitochondrial membrane potential.

**Figure 4.**
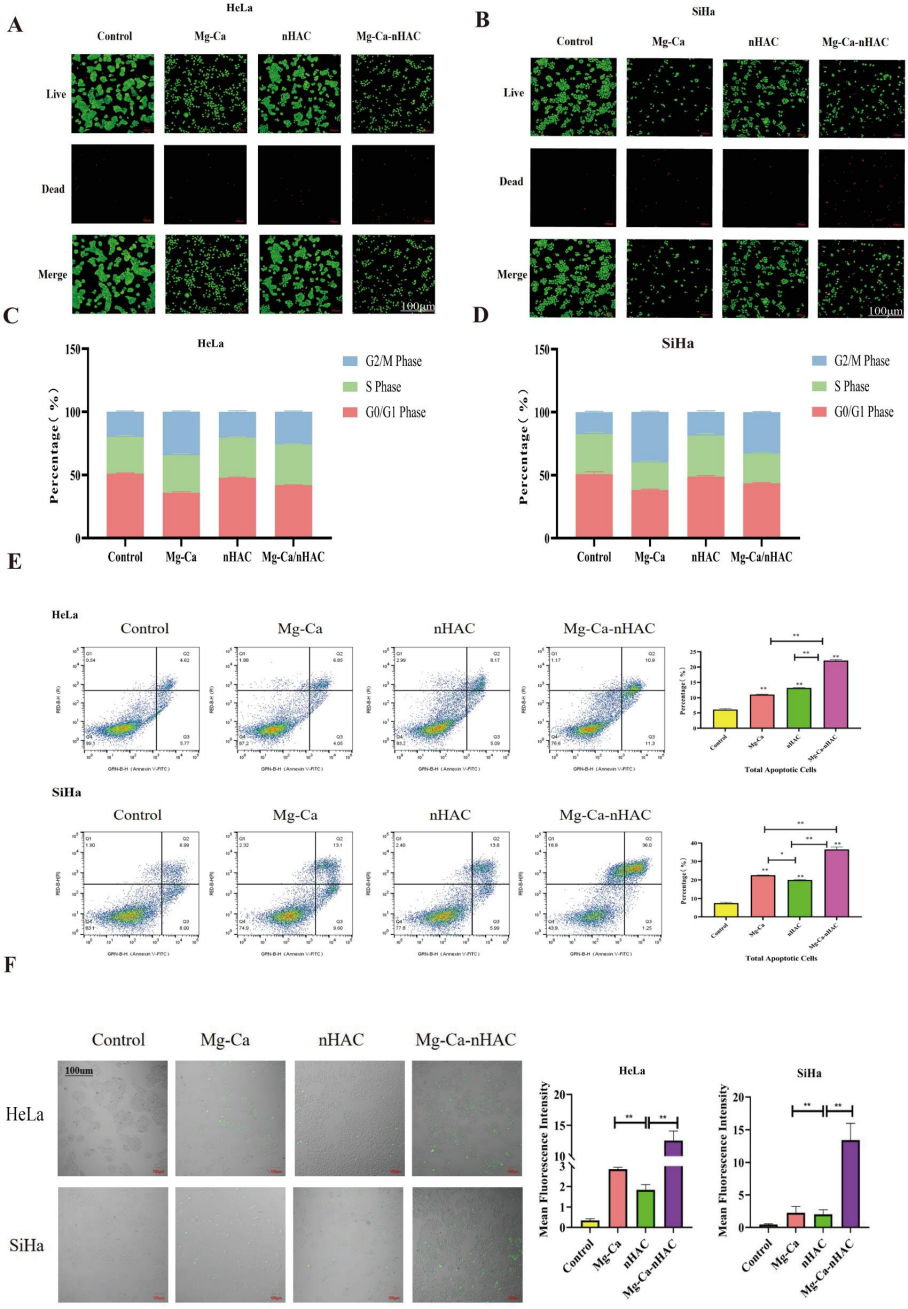
*In vitro* anti-tumor assays. (**A, B**) The number of live and dead cells in the different treatment groups was detected by live-dead cell staining and the fluorescence intensity was quantified (scale bar: 100 μm, green: live cells, red: dead cells). (**C**) Detection of cell cycle of HeLa cells in different treatment groups by flow cytometry. (**D**) Detection of cell cycle of SiHa cells in different treatment groups by flow cytometry. (**E**) Detection of apoptosis in HeLa cells/SiHa cells of different treatment groups by flow cytometry (*P < 0.05, **P < 0.01). (**F**) Detection of ROS levels in different treatment groups by flow cytometry (scale bar: 100 μm, **P < 0.01).

As shown in Figure 4A and B, the results of live and dead cell staining showed that in the Mg-Ca group and the Mg-Ca-nHAC composite scaffolds group, the expression of green fluorescence was relatively low, and the red fluorescence was relatively high, especially in Mg-Ca-nHAC composite scaffolds group, which indicated that there was a significant inhibitory effect on cervical cancer, which was consistent with the results of the CCK8 experiment (green: live cells, red: dead cells). Figure 4C showed the effect of different treatment groups on HeLa cell cycle and the results showed that nHAC, and Mg-Ca-nHAC composite scaffolds inhibited the cell growth mainly in the G2/M phase, in the late and interphase of cytokinesis. However, Mg-Ca group inhibited cell proliferation mainly in the S phase, this is the DNA replication phase. The effect of different treatment groups on SiHa cell cycle was detected by flow assay as shown in Figure 4D, and the results indicated that nHAC group inhibited cell growth mainly in the G2/M phase, which is in the late cell division and interphase. Mg-Ca and Mg-Ca-nHAC composite scaffolds groups inhibited cell growth mainly in the S phase, which is in the cell replication phase. It may be that degradation product Mg^2+^ inhibits tumor proliferation through DNA repair mechanisms. Apoptosis is a crucial factor resulting in cell proliferation inhibition [29, 30]. Besides, apoptosis was detected by flow cytometry and the experimental results showed that the Mg-Ca-nHAC composite scaffolds inhibited cell growth most obviously and there was a significant difference with the comparison between groups. In addition, the Mg-Ca group and nHAC group also showed significant differences in apoptosis-promoting effects when compared to the control group as shown in Figure 4E. Mg has been shown to interfere with cancer formation through two mechanisms: it modulates inflammatory conditions and free radical radical-induced oxidative stress induced by free radicals, both of which can lead to DNA damage and cancer. ROS in tumor cells can induce tumor cell apoptosis, which is the mechanism of cancer suppression by many anti-tumor drugs [31–33]. Figure 4F showed that ROS production was similarly different between the two cell types: both experimental treatment groups exhibited significant ROS expression, with the highest in the Mg-Ca-nHAC group. The results showed that Mg-Ca-nHAC induced ROS to induce apoptosis of cervical cancer cells through redox equilibrium.

**Figure 5.**
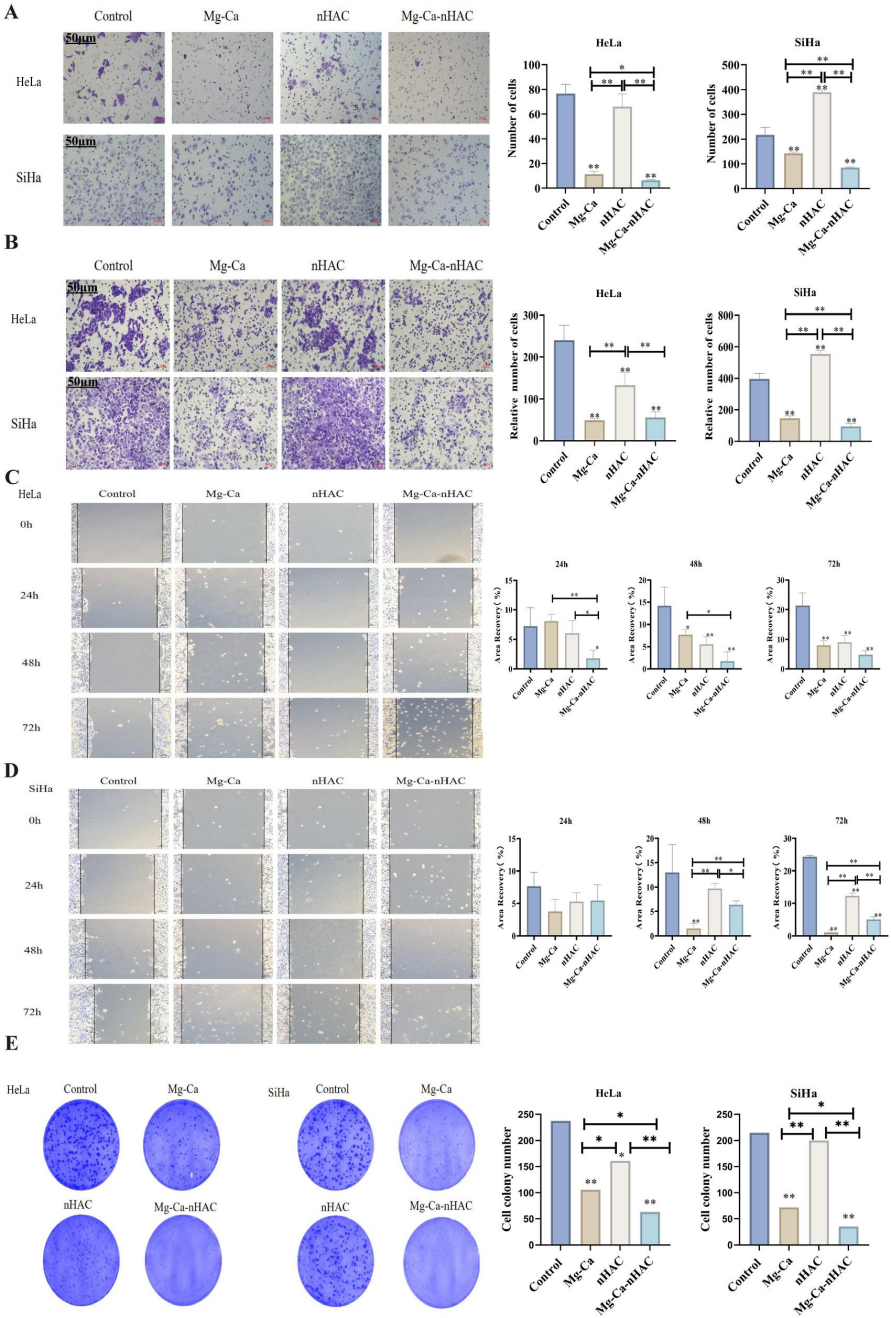
*In vitro* anti-tumor assays. (**A**) The migration of HeLa and SiHa cells was observed by microscopic photography and quantitative analysis (scale bar: 50 μm, *P < 0.05, **P < 0.01). (**B**) The invasion of HeLa and SiHa cells was observed by microscopic photography and quantitative analysis (scale bar: 50 μm, **P < 0.01). (**C, D**) The migration of HeLa and SiHa cells was observed by microscopic photography and quantitative analysis through scratch experiments (*P < 0.05, **P < 0.01). (**E**) Plate cloning to further test the proliferative clonogenic capacity of cells (*P < 0.05, **P < 0.01).

The proliferative capacity of tumor cells was closely related to their metastatic capacity. Tumor cells with high proliferation rates tend to have stronger metastatic potential [34, 35]. On the one hand, the proliferation process may generate subpopulations of tumor cells with higher invasiveness and migration ability. On the other hand, rapid proliferation will lead to changes in the internal environment of the tumor tissue, such as an increase in the secretion of angiogenic factors to meet the nutrient requirements of tumor cell growth. These angiogenic factors also helped tumor cells to enter the blood circulation, and then metastasize to distant places [36–38]. The previous study showed that the composite scaffolds had a significant inhibitory effect on the growth of cervical cancer cells, in order to further verify whether the treatment groups inhibit cell growth and promote apoptosis through invasion and metastasis. This study further detected the results of cell migration by Transwell. Since Cellular migration is closely related to cancer metastasis [39], this study speculated whether Mg-Ca-nHAC composite scaffolds promote apoptosis and inhibit growth by inhibiting tumor invasion and metastasis. The experimental results are shown in Figure 5A: Compared with the control group, cell migration was significantly inhibited in the Mg-Ca-nHAC composite scaffolds group, followed by the Mg-Ca group. Compared with the nHAC group and the intergroup, there was also a significant inhibitory effect (*P < 0.05, **P < 0.01). The results of the Transwell cell invasion assay are shown in Figure 5B: compared with the control group, the Mg-Ca-nHAC composite scaffolds group significantly inhibited cell invasion, followed by the Mg-Ca group (**P < 0.01). In addition to the control group, significant inhibitory effects with statistical significance were also observed when comparing the Mg-Ca-nHAC group with the nHAC group and among other groups. In addition, scratch test was further used to detect the invasive ability of the HeLa/SiHa cells at 24, 48 and 72 h (Figure 5C and D), and the experimental results remained consistent with the above. The results showed that at 24, 48 and 72 h, HeLa cells in the Mg-Ca-nHAC composite scaffold group exhibited the least migration, followed by those in the Mg-Ca and nHAC groups. Meanwhile, the results showed that there was no statistically significant difference in SiHa cell migration rate between groups at 24 h cell phase. However, at 48 and 72 h, Mg-Ca group had the least cell migration rate, followed by Mg-Ca-nHAC composite scaffolds and nHAC groups. This may be due to the fact that SiHa cells are less sensitive to Mg-Ca-nHAC composite scaffolds than HeLa cells, mainly due to the inhibitory effect exerted by Mg-Ca alloys. These results indicated that Mg-Ca-nHAC composite scaffolds have the potential to prevent cancer metastasis. According to the above results, Mg-Ca and nHAC materials synergistically produce an anti-tumor effect. This study further verified cell clonogenic proliferation ability using plate cloning assay, with results shown in Figure 5E. Microscopic observation and quantitative analysis revealed statistically significant differences between all treatment groups and the control group. Intergroup comparisons indicated that the Mg-Ca-nHAC composite scaffold group exhibited the weakest clonogenic ability, followed by the Mg-Ca group and nHAC group.

#### Transcription analysis

**Figure 6.**
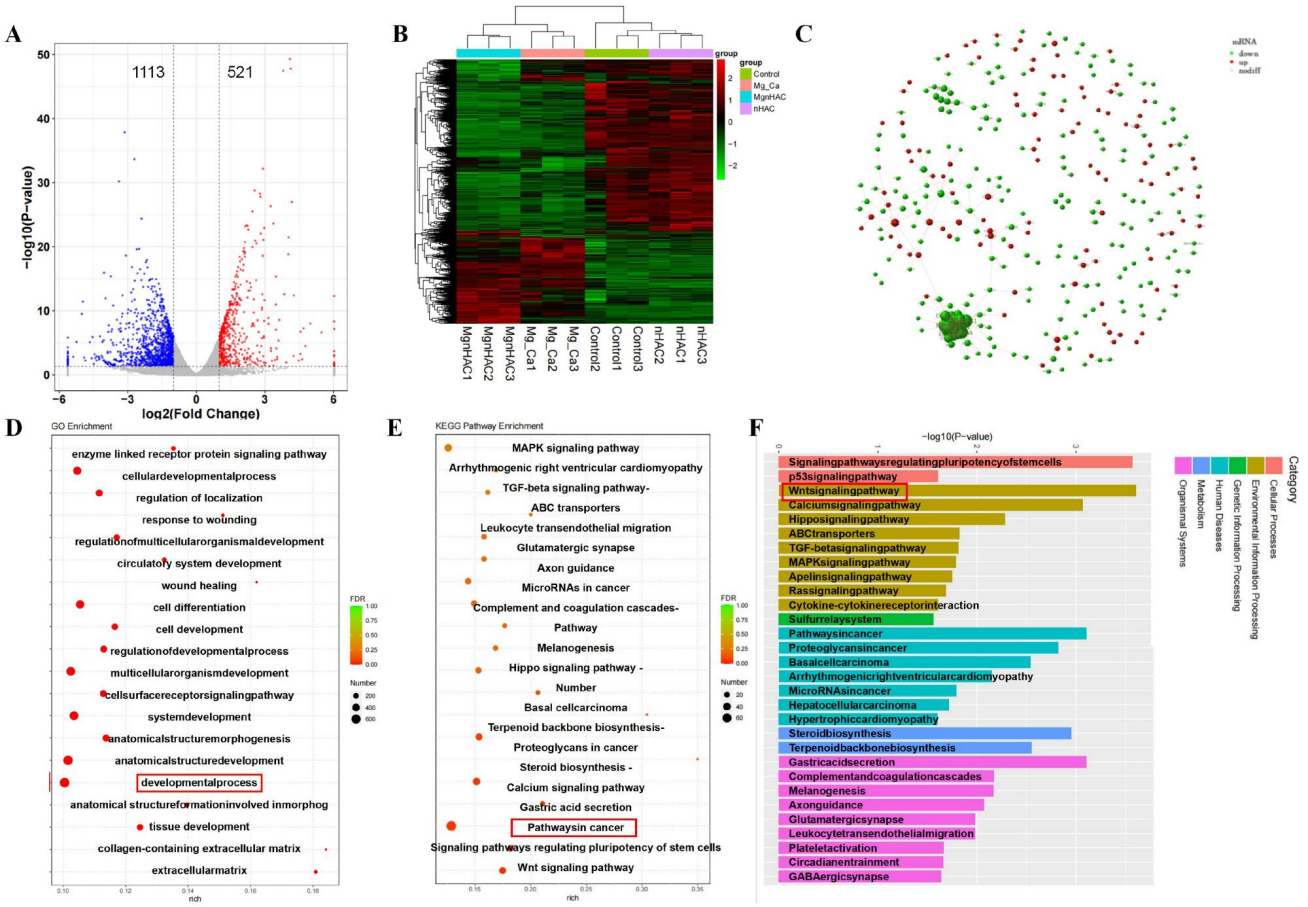
*In vitro* anti-tumor assays. Transcriptome analysis was exhibited by (**A**) volcano plot, (**B**) heatmap, (**C**) PPI chart, (**D**) pathways enrichment based on GO enrichment analysis was showed by bubble chart plot, (**E**) pathways enrichment of DEGs based on KEGG enrichment analysis was showed by bubble chart plot. (**F**) Pathways enrichment of DEGs based on KEGG enrichment analysis was showed by histogram.

In order to verify the pathway by which the Mg-Ca composite scaffolds inhibited the invasive and metastatic growth mechanisms of tumor, this study used bioinformatics transcriptional analysis. Transcriptomic analysis results presented in Figure 6 revealed gene expression differences between groups and suggested the potential anti-tumor activity of the Mg-Ca-nHAC composite scaffolds. Figure 6A demonstrated that compared to the Mg-Ca composite scaffolds group, the control group exhibited 1113 downregulated genes and 521 upregulated genes. Figure 6B showed that the heatmap clearly indicated significant enrichment differences between groups, with notable disparities in gene expression profiles observed between the Mg-Ca group and the Mg-Ca-nHAC composite scaffolds group, as well as between the control group and the nHAC group. This suggested that nHAC incorporation may influence scaffold function by regulating gene expression. Figure 6C revealed that protein encoded by the differentially expressed genes formed interaction modules within the PPI network, with core nodes providing clues for identifying key targets in anti-tumor mechanisms. As indicated by the GO enrichment bubble chart, Figure 6D showed that these differentially expressed genes were enriched in biological processes related to tumor progression, such as cell development. KEGG enrichment analysis in Figure 6E and F (bubble diagram and histogram) further confirmed that the differentially expressed gene was primarily enriched in tumor-related pathways, with the Wnt signaling pathway serving as the core. These findings indicated that the Mg-Ca-nHAC composite scaffolds may exert potential anti-tumor effects against invasion and metastasis by regulating gene expression and modulating the Wnt signaling pathway.

#### Verify the relevant signal pathway

**Figure 7.**
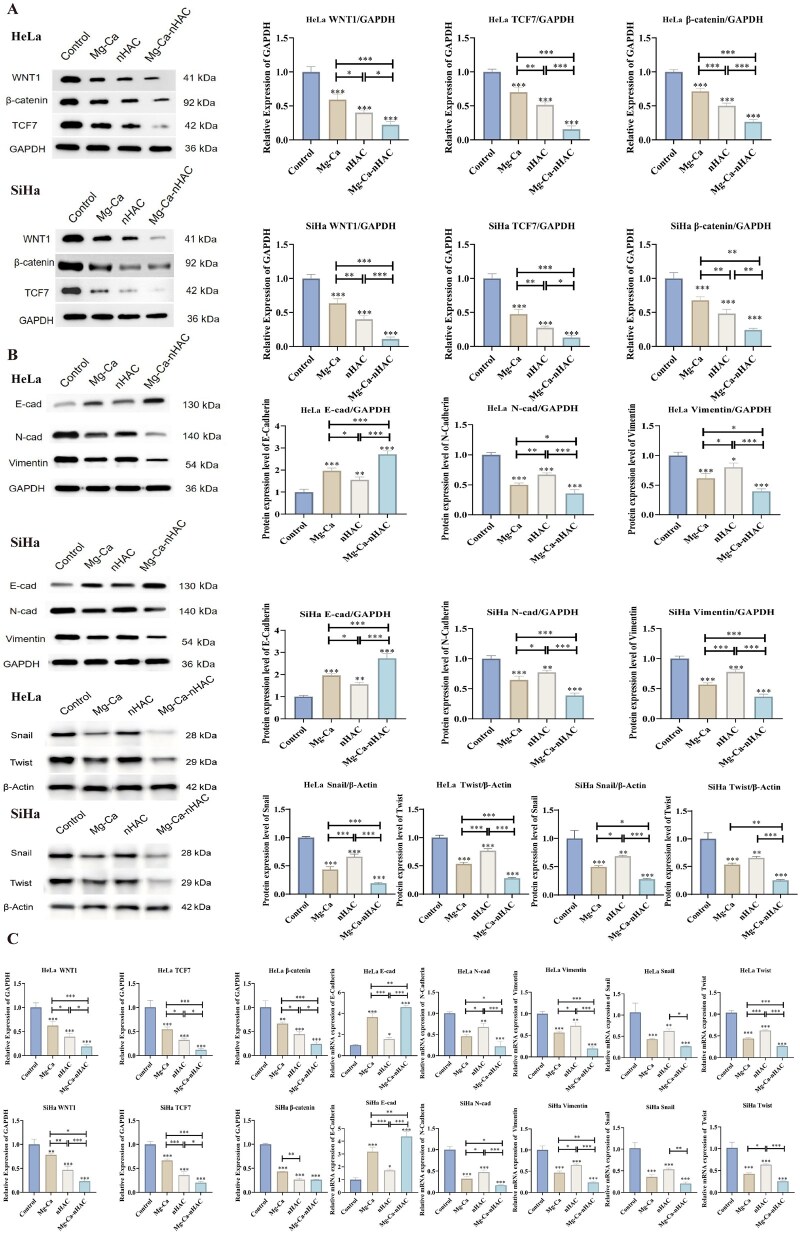
*In vitro* anti-tumor assays. (**A, B**) The expression of protein levels (Wnt1, β-catenin, TCF7, E-cadherin, N-cadherin, Vimentin, Snail, Twist) in different treatment groups was detected by Western blot assay (*P < 0.05, **P < 0.01,***P < 0.001). (**C**) The expression of gene levels (Wnt1, β-catenin, TCF7, E-cadherin, N-cadherin, Vimentin, Snail, Twist) in different treatment groups was detected by PCR experiment (*P < 0.05, **P < 0.01,***P < 0.001).

According to the above results of transcriptional analysis (Figure 6), the Mg-Ca composite scaffolds exerted their anti-tumor effects mainly through the Wnt signaling pathway, which was an important signaling pathway for cell cycle regulation, inflammation and neoplasia [40]. Aberrant Wnt signaling has been associated with the development of colorectal, breast, lung, oral, cervical and hematopoietic malignancies [41]. In classical Wnt/β-catenin signaling pathway, when the Wnt ligand binds to the receptor on cell, it reduces degradation of intracellular β-catenin protein and increases the accumulation of intracellular β-catenin protein and the increased amount of β-catenin can enter the nucleus and bind to the TCF/LEF1 factor to activate the expression of the target gene [42]. TCF7 acted as an oncogene in colorectal cancer [43], gastric cancer [44] and glioma [45]. TCF7 is a key component of Wnt signaling. Several drugs, genes and non-coding RNAs have been reported to play important roles in human diseases by regulating TCF7 in the Wnt pathway [46]. In this study, Western blot and PCR were used to detect the expression of related gene and protein. As shown in Figure 7A, the result indicated that Mg-Ca-nHAC composite scaffolds showed a significant decrease in Wnt, β-catenin and TCF7 protein expression levels in HeLa/SiHa cells compared to the control group and other treatment groups. The results suggested that the Mg-Ca-nHAC composite scaffolds exerted anti-tumor effects by inhibiting Wnt/β-catenin/TCF7 signaling pathway. EMT is considered a key factor in tumor invasion and metastasis [47], and it is characterized by the absence of epithelial cell markers (e.g. cytokeratins and E-cadherin) and upregulation of the expression of mesenchymal cell markers (e.g. N-cadherin, Vimentin and Snail, Twist) [48]. As shown in Figure 7B, the results showed that E-cadherin was significantly up-regulated and N-cadherin, Vimentin, Snail and Twist protein expression levels were significantly down-regulated in the Mg-Ca-nHAC composite scaffolds group, followed by the Mg-Ca and nHAC groups. It also further indicated that the Mg-Ca-nHAC composite scaffolds had the most significant effect on inhibiting invasive metastasis of cervical cancer, followed by the Mg-Ca and nHAC groups, which was consistent with the results in Figure 5A and B. Furthermore, the consistency between the PCR results (Figure 7C) and the Western blot data provided additional verification for the aforementioned conclusion. Based on the above results, Mg-Ca-nHAC composite scaffolds exerted anti-tumor effects by inhibiting EMT through the Wnt/β-catenin/TCF7 signaling pathway, which needs to be further explored.

**Figure 8.**
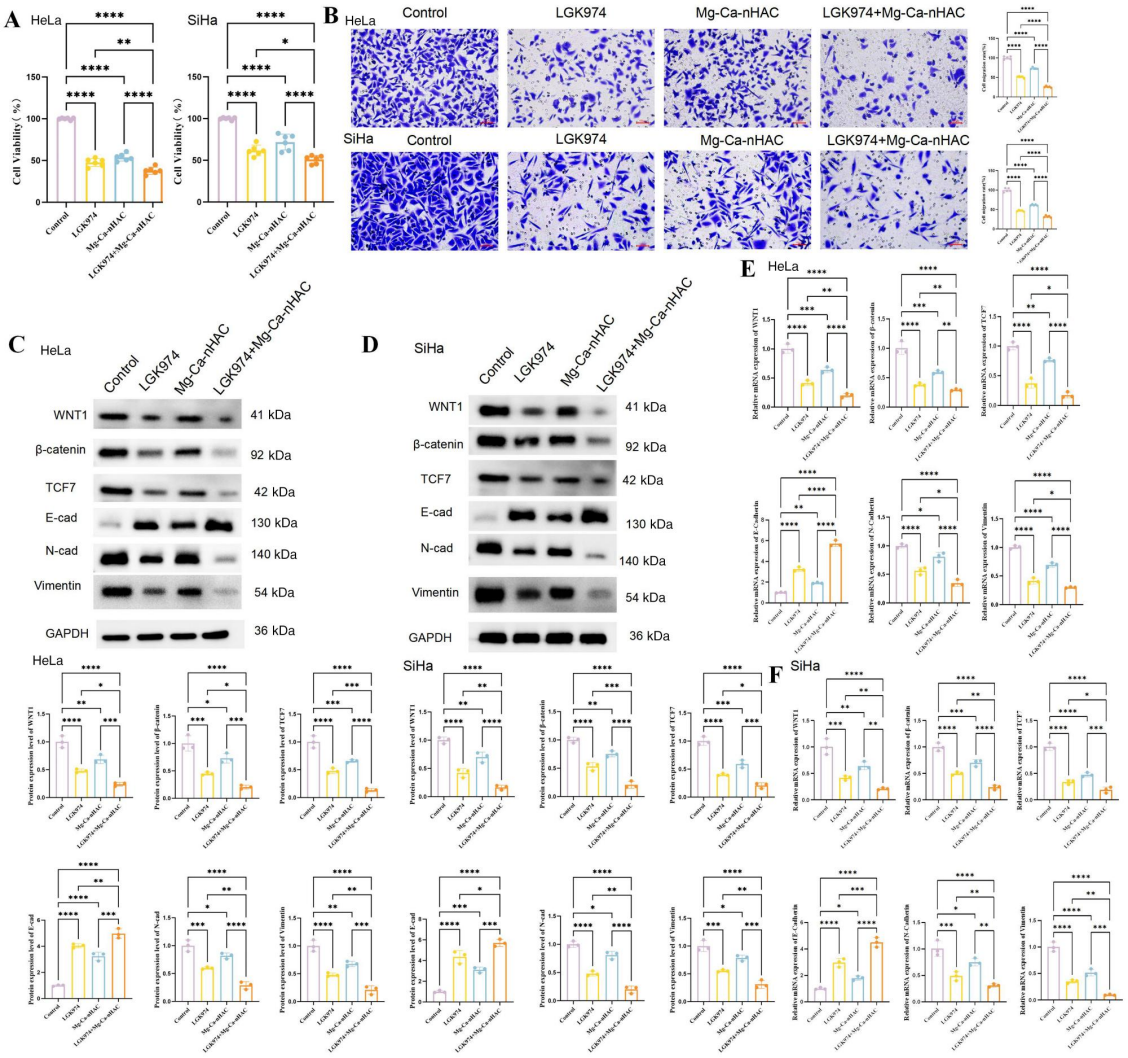
*In vitro* anti-tumor assays. (**A**) In different cell lines (HeLa/SiHa), the cell proliferation activity of different treatment groups was detected at 24 h through the CCK8 experiment (*P < 0.05, **P < 0.01,****P < 0.0001). (**B**) The cell migration detection of HeLa and SiHa cells was observed by microscopic photography and quantitative analysis (scale bar: 100 μm, ****P < 0.0001). (**C, D**) The expression of protein levels (Wnt1, β-catenin, TCF7, E-cadherin, N-cadherin, Vimentin) in different treatment groups was detected by Western blot assay (*P < 0.05, **P < 0.01,***P < 0.001, ****P < 0.0001). (**E, F**) The expression of gene levels (Wnt1, β-catenin, TCF7, E-cadherin, N-cadherin, Vimentin) in different treatment groups was detected by PCR experiment (*P < 0.05, **P < 0.01,***P < 0.001,****P < 0.0001).

To further validate the mechanisms by which Mg-Ca-nHAC exerts its anti-tumor effect, we conducted a reverse validation. According to the results shown in Figure 8A, CCK-8 assay results showed that LGK974 and Mg-Ca-nHAC had different effects on the viability of HeLa and SiHa cells. In HeLa cells, LGK974 significantly inhibited cell viability (***P < 0.001), and Mg-Ca-nHAC was also effective (**P < 0.01). However, the combined treatment (LGK974+Mg-Ca-nHAC) exhibited the strongest inhibitory effect (****P < 0.0001), suggesting a synergistic effect. In SiHa cells, Mg-Ca-nHAC had a more significant inhibitory effect (***P < 0.001), while LGK974 had a weaker effect (*P < 0.05). The combined treatment had an effect comparable to that of Mg-Ca-nHAC and did not show synergistic effects. The results indicated that the regulation of cervical cancer cell viability by the two groups was cell type-dependent, with HeLa cells being more sensitive to combined treatment. Further cell migration experiments were conducted (Figure 8B). Migration assay results showed that LGK974 and Mg-Ca-nHAC significantly inhibited the migration ability of HeLa and SiHa cells. In HeLa cells, both LGK974 group (****P < 0.0001) and Mg-Ca-nHAC group (****P < 0.0001) significantly reduced cell migration rates, while the combined treatment (LGK974+Mg-Ca-nHAC) exhibited the most pronounced inhibitory effect (****P < 0.0001), suggesting a potential synergistic effect. In SiHa cells, both single treatments (LGK974 and Mg-Ca-nHAC) significantly inhibited migration (****P < 0.0001), but the combined treatment did not further enhance the effect compared to single treatments. The results indicated that both treatment groups effectively inhibited the migration of cervical cancer cells, with a more pronounced synergistic inhibitory effect on HeLa cells. Western blot results (Figure 8C and D) showed that LGK974 and Mg-Ca-nHAC treatment significantly regulated the Wnt/β-catenin pathway and EMT marker expression in HeLa and SiHa cells. In HeLa cells, LGK974 treatment significantly downregulated Wnt, β-catenin and TCF7 expression (***P < 0.001), upregulated E-cadherin (**P < 0.01), and inhibited N-cadherin and Vimentin (****P < 0.0001). Combined treatment (LGK974+Mg-Ca-nHAC) further enhanced the inhibitory effects on β-catenin and TCF7. In SiHa cells, both treatments (LGK974 and Mg-Ca-nHAC) significantly reduced β-catenin, TCF7 and Vimentin expression (***P < 0.001), and combined treatment had the most significant inhibitory effect on Wnt (****P < 0.0001). The data from both groups collectively indicated that LGK974 and Mg-Ca-nHAC can inhibit cervical cancer by synergistically inhibiting the Wnt/β-catenin pathway and reversing the EMT process. As shown in Figure 8E and F, PCR result showed that LGK974 and Mg-Ca-nHAC treatment significantly affected the expression of EMT-related gene in HeLa and SiHa cells. In HeLa cells, LGK974 treatment significantly downregulated β-catenin, TCF7 and Wnt expression (***P < 0.001), upregulated E-cadherin (**P < 0.01) and inhibited N-cadherin and Vimentin (****P < 0.0001). LGK974+Mg-Ca-nHAC further enhanced the inhibitory effects on β-catenin and TCF7. In SiHa cells, both LGK974 and Mg-Ca-nHAC alone significantly inhibited Wnt expression, with the most pronounced effect observed in the combined treatment group (LGK974+Mg-Ca-nHAC) (****P < 0.0001). The data from both groups collectively indicated that LGK974 and Mg-Ca-nHAC could inhibit cervical cancer cells by inhibiting the Wnt/β-catenin/TCF7 pathway and regulating the expression of EMT markers.

### *In vivo* experiment

**Figure 9.**
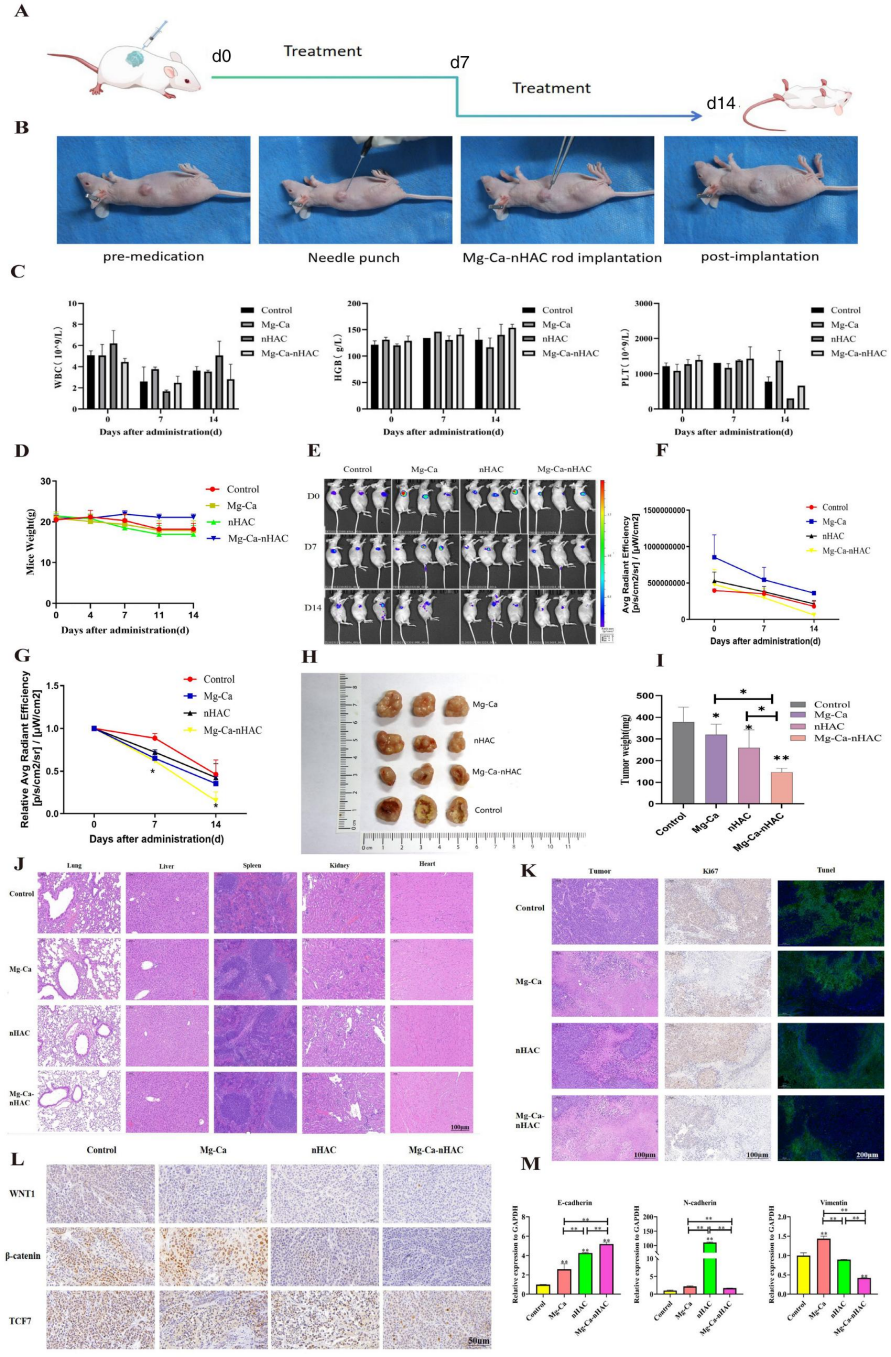
*In vivo* level anti-tumor assay. (**A**) Scheme of subcutaneous HeLa-Luc mouse tumor model for different treatment groups. (**B**) Pre-implantation, post-implantation course of Mg-Ca rods and nHAC materials after establishment of a mouse tumor model. (**C**) Routine blood tests for routine white blood cell, hemoglobin and platelet counts were performed on mice 0, 7 and 14 days after implantation of the materials. (**D**) Observe the changes in body weight of mice under the effect of different treatment groups at each time point. **(E–G)** Mice from different treatment groups were photographed and analyzed for fluorescence intensity *in vivo* imaging at 0, 7 and 14 days. (**H–I**) Tumors were sampled and photographed and tumor volumes were measured in mice from different treatment groups at 14 days (*P < 0.05, **P < 0.01). (**J**) HE staining microscopic images of vital organs in different treatment groups after tumor sampling (scale bar: 100 μm). (**K**) Microscopy images of HE, Tunel and Ki67 stained (HE and Ki67 scale bar: 100 μm, Tunel scale bar: 200 μm). (**L**) Immunohistochemical staining for Wnt1, β-catenin, TCF7 after sampling (scale bar: 50 μm). (**M**) Detection of E-cadherin, N-cadherin, Vimentin gene expression level by PCR (**P < 0.01).

Next, this study further tested it *in vivo*. As shown in Figure 9A, a mouse subcutaneous tumor model was first constructed, and changes in mouse body weight and tumor volume were observed at 0, 7 and 14 days. As shown in Figure 9B, the materials were implanted in the following treatment (antibiotics should be injected continuously for 3 days after implantation to prevent infection, and Mg rod materials should be weighed before implantation). In this experiment, Mg rod implantation was used *in vivo*, and individual nHAC was injected in accordance with the extraction solution (Mg-Ca alloy rods-1×7 mm size). In order to evaluate the biosafety *in vivo*, blood samples were taken every week (0, 7 and 14 days) for routine blood tests, including white blood cell, hemoglobin and platelet counts. The blood routine results were shown in Figure 9C, and there was no significant difference between the treatment group and the control group, which was within the normal value range, indicating that the materials in the treatment group had certain biosafety. As illustrated in Figure 9D, this study observed the weight of mice at different time points and found that there was no significant change in the weight of mice in the treatment group. It indicates that Mg-Ca-nHAC group therapy has no significant side effects and is biologically safe. Further in this study, live imaging images of mice were taken and fluorescence intensity was analyzed as shown in Figure 9E and F. The results indicated that the fluorescence intensity in the Mg-Ca-nHAC group, nHAC group and Mg-Ca group all decreased significantly over time. As shown in Figure 9G, the Mg-Ca-nHAC group also had the lowest fluorescence intensity, while there was no significant difference between the nHAC group and Mg-Ca group. Tumor tissues were removed by executing the mice on 14 days. This study performed tumor sampling at 14 days and found that the Mg-Ca-nHAC composite scaffolds group had the smallest tumor weight compared with the other groups, followed by the nHAC group and Mg-Ca group, and there was a statistical difference. It indicated that the Mg-Ca-nHAC group significantly inhibited tumor growth (Figure 9H and I). At the end of treatment, major organs of the tumor-bearing mice were stained with HE to evaluate the biocompatibility of Mg-Ca-nHAC group. As depicted in Figure 9J, there were no adverse effect on the lung, liver, spleen, kidney in Mg-Ca-nHAC group compared to the control group. In particular, no nodules were found in the lung tissue, indicating that the tumor tissue had not metastasized. It suggests that the biocompatibility and biosafety of Mg-Ca-nHAC group neither cause organ damage nor promote tumor cell metastasis. Ki67 is a marker to test the active proliferation of tumor cells, and we could see from the staining results that Mg-Ca-nHAC group had the lowest expression of Ki67, and the tumor cells were not in the stage of active proliferation, which also indicated that Mg-Ca-nHAC group significantly inhibited the growth of cervical cancer (as was shown in Figure 9K). Tunel staining, a terminal deoxynucleotidyl transferase-mediated dUTP nick end labeling assay, is a commonly used technique for detecting DNA fragmentation in apoptosis [49]. The results indicated that Mg-Ca-nHAC group significantly inhibited the growth of cervical cancer. Subsequently, the tumor tissues were processed for immunohistochemical staining to detect the expression of relevant proteins/genes. As shown in Figure 9L, Mg-Ca-nHAC group showed lowest levels of Wnt, β-catenin and TCF7 protein expression compared to the control group. This was followed by the nHAC group and Mg-Ca group, with the same results as the *in vitro* experiments. Mg-Ca-nHAC group inhibited the growth, invasion and metastasis of the HeLa cells by activating the Wnt/β-catenin/TCF7 signaling pathway. The present study further verified the expression of the relevant gene levels, as shown in Figure 9M, and the results were also consistent with the *in vitro* PCR experiments. In a word, Mg-Ca-nHAC composite scaffolds exerted anti-tumor effects by activating the Wnt/β-catenin/TCF7 signaling pathway to inhibit EMT in cervical cancer.

**Figure 10.**
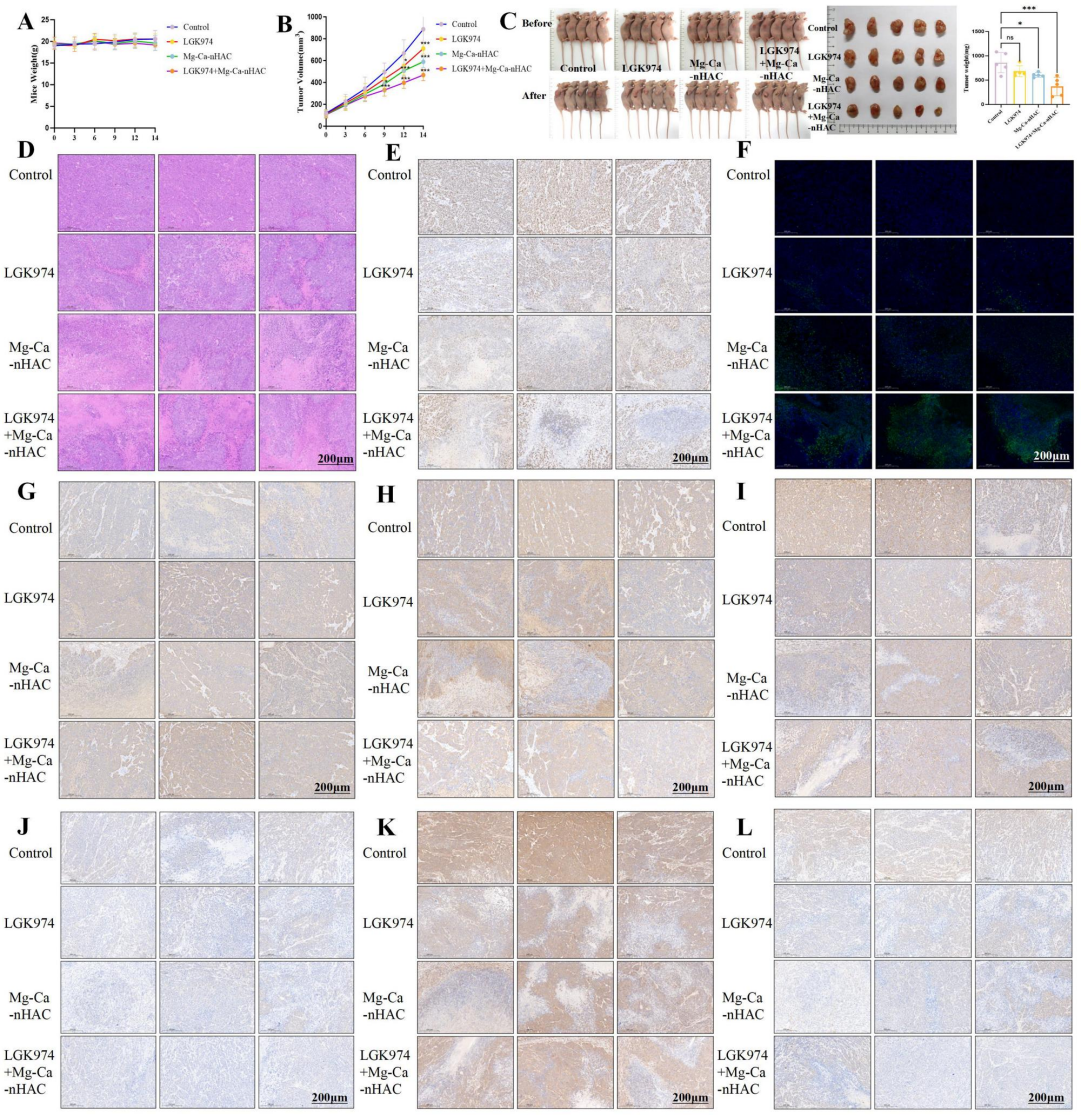
*In vivo* level anti-tumor assay. (**A**) Observe the changes in body weight of mice under the effect of different treatment groups at each time point. (**B**) Changes in mice tumor volume at each time point for the different treatment groups. (**C**) Observe photographs of mice in each group administered the drug sequentially, and perform quantitative analysis of the average tumor weight in different groups by removing tumor tissue after 14 days and taking photographs. (**D–F**) Perform HE, Ki67 and Tunel staining on tumor tissue (scale bar: 200 μm). (**G–I**) Immunohistochemistry staining of EMT markers (E-cadherin, N-cadherin and Vimentin, scale bar: 200 μm). (**J–L**) Immunohistochemistry staining of Wnt, β-catenin, TCF7 (scale bar: 200 μm).

As illustrated in Figure 10A, this study observed the weight of mice at different time points and found that there was no significant change in the weight of mice in the treatment group. It indicated that LGK974 and Mg-Ca-nHAC group therapy have no significant side effects and were biologically safe. Figure 10B showed the changes in average tumor volume at different time points across different groups. The results indicated that compared with the control group, the tumor volumes in all three treatment groups were smaller. LGK974 group showed significant therapeutic effects 12 days after treatment, while Mg-Ca-nHAC group and LGK974+Mg-Ca-nHAC group, especially LGK974+ Mg-Ca-nHAC group, began to show significant therapeutic effects as early as the ninth day after treatment, with statistically significant differences (*P < 0.05,***P < 0.001). The average anti-tumor efficacy of the experimental groups was ranked as follows: LGK974+Mg-Ca-nHAC>Mg-Ca-nHAC>LGK974>control group. Figure 10C shows photographs of mice in each group taken before and after drug administration on Day 14, and tumor in each group was photographed after tissue collection. The average tumor weight of different groups on Day 14 was measured. The results showed that compared with the control group, the tumor weights of all treatment groups were smaller, with the most significant difference observed in Mg-Ca-nHAC group and LGK974+Mg-Ca-nHAC group, and the difference was significant (*P < 0.05,***P < 0.001). The average anti-tumor efficacy of each group in the experiment was ranked as LGK974+Mg-Ca-nHAC>Mg-Ca-nHAC >LGK974>control group. This study performed HE staining on tumor tissues, with results shown in Figure 10D. In the control group, tumor cells were densely arranged, with frequent mitotic figures, significant cellular atypia, rare necrotic areas and rich vascular stroma—indicating a highly malignant proliferative state. Tumor cells density in LGK974 inhibitor group was decreased, with small patchy necrotic areas visible locally, reduced mitotic figures, mild proliferation of interstitial fibrous tissue and inflammatory cell infiltration (predominantly lymphocytes). In Mg-Ca-nHAC, extensive necrotic foci appeared in the tumor tissue, with prominent fibrous encapsulation around the necrotic areas, increased collagen deposition in the stroma, loosely arranged tumor cells and reduced atypia. The LGK974+Mg-Ca-nHAC group exhibited the most significant disruption of tumor structure, with extensive necrotic areas covering more than 50% of the tumor volume, residual tumor cells distributed in small nests, significantly enhanced interstitial fibrosis and inflammatory infiltration, and occasional calcification foci (possibly related to the deposition of nHAC). As shown in Figure 10E, Ki67 is a nuclear protein associated with cell proliferation and is widely used to assess the proliferative activity of tumor cells. A higher proportion of positive cells (brown) indicates more active tumor cell proliferation, suggesting higher degree of malignancy. The results showed that compared with the control group, the proportion of Ki67-positive cells in tumor tissues was significantly reduced in all treatment groups, with LGK974+Mg-Ca-nHAC group< Mg-Ca-nHAC group< LGK974 group< control group, suggesting that the treatment groups may inhibit tumor growth by suppressing proliferation. Tunel staining is a classic experimental technique used to detect cell apoptosis by identifying apoptotic cells through the labeling of the 3′-hydroxyl ends of DNA fragments. Figure 10F shows that compared with the control group, the proportion of Tunel-positive cells (green) in the tumor tissues of each treatment group was significantly increased, with LGK974+Mg-Ca-nHAC>Mg-Ca-nHAC>LGK974>control group, suggesting that the drug may inhibit tumor growth by inducing apoptosis. Furthermore, this study performed immunohistochemical staining of EMT markers (E-cadherin, N-cadherin and Vimentin). The results indicated that E-cadherin, a member of the cadherin family, is primarily expressed in epithelial cells and maintains intercellular junctions. N-cadherin is typically expressed in mesenchymal cells and participates in cell migration. Changes in their expression are often associated with EMT, where epithelial cells transform into mesenchymal cells, promoting tumor invasion and metastasis. The reduction in E-cadherin indicates a loss of intercellular adhesion, promoting metastasis. Increased levels of N-adhesin promote cellular migratory capacity. Decreased E-cadherin and increased N-cadherin are hallmarks of EMT. Figure 10G and H shows that compared with the control group, the proportion of E-cadherin-positive cells in tumor tissues significantly increased in all treatment groups (LGK974+Mg-Ca-nHAC>Mg-Ca-nHAC≈LGK974>control group), while the proportion of N-cadherin-positive cells decreased (LGK974+Mg-Ca-nHAC<Mg-Ca-nHAC≈LGK974<control), suggesting that the treatment groups may inhibit tumor growth by suppressing EMT. Vimentin is a marker protein of mesenchymal cells that participates in EMT and promotes tumor cell migration and metastasis. Figure 10I shows that compared with the control group, the proportion of Vimentin-positive tumor tissues decreased locally in each treatment group (LGK974+Mg-Ca-nHAC group < Mg-Ca-nHAC group< LGK974 group< control group), suggesting that the treatment groups may inhibit tumor growth by suppressing EMT. Figure 10J–L shows immunohistochemical staining for Wnt, β-catenin and TCF7, respectively. WNT1 is a ligand of the Wnt pathway. Activation of the pathway leads to the accumulation of β-catenin in the cell nucleus. β-catenin is a key molecule in the Wnt pathway. Under normal conditions, it is degraded by a complex in the cytoplasm. Upon pathway activation, it enters the nucleus, promoting cell proliferation and EMT. Figure 10J and K shows that compared with the control group, the proportion of Wnt and β-catenin-positive tumor tissues decreased in all treatment groups, suggesting that the drugs may inhibit tumor growth by suppressing EMT. TCF7 is a downstream transcription factor of the Wnt/β-catenin signaling pathway. After binding to nuclear β-catenin, it activates oncogenes (such as c-Myc and Cyclin D1), driving cell proliferation and stem cell characteristics. High expression is associated with tumor invasiveness, chemotherapy resistance and poor prognosis. Figure 10L results show that compared with the control group, the proportion of TCF7-positive tumor tissue decreased in all treatment groups (LGK974+Mg-Ca-nHAC group< Mg-Ca-nHAC group< LGK974 group< control group), suggesting that the Mg-Ca-nHAC group may inhibit tumor growth by suppressing EMT.

## Discussion

Mg and its alloys have gained widespread recognition as promising biodegradable materials, particularly in the field of orthopedic implants [50, 51]. Mg is spontaneously degraded to Mg(OH)_2_ and H_2_ under physiological or buffered conditions due to its relatively low standardized reduction potential (−2.37VSHE) [52]. Above all, our work suggested that Mg-based implant composite scaffolds may be a viable pathway to achieve safe and effective H_2_ therapy, which may be combined with other therapeutic strategies such as cancer immunotherapy in future clinical trials. The tumor microenvironment was usually weakly acidic, with low oxygen levels and high ROS levels, as well as immunosuppressive [53, 54]. Mg-based composite scaffolds achieve excellent anti-tumor effects by altering the tumor microenvironment.

This work aimed to assess Mg-based materials as a groundbreaking localized treatment approach for suppressing metastatic cancer development and spread. This experiment systematically characterized the morphology, composition and surface properties of Mg-Ca-nHAC composite materials using SEM, EDS and AFM. The results confirmed that unprotected Mg-Ca alloys exhibit surface oxidation issues (Figure 1A), highlighting the necessity of surface modification. The application of nHAC coating (Figure 1C) is an effective strategy to address this challenge, mainly by physically isolating the substrate from contact with the environment, thereby significantly inhibiting the oxidation and corrosion rates. The nHAC coating exhibits distinct nanoscale porous structural features (Figure 1B and C). This structure mimics the extracellular matrix of natural bone, providing an ideal microenvironment for cell adhesion, proliferation and nutrient exchange, while also possessing the potential to serve as a slow-release carrier for bioactive molecules, which is crucial for promoting osseointegration. EDS analysis (Figure 1D–I) clearly validated the key chemical components of the coating, particularly the detection of abundant P and Ca elements, directly confirming the successful incorporation of nano-hydroxyapatite. Hydroxyapatite, as the primary inorganic mineral of bone, confers the composite material with excellent bioactivity, osteoconductivity and potential osteoinductivity. Additionally, the C and O elements in the coating primarily originate from the collagen matrix. The presence of this organic phase further enhances the material’s biocompatibility and makes its composition more akin to natural bone tissue. AFM characterization (Figure 1J) further revealed that the coating surface exhibits overall good flatness and uniformity. This surface characteristic is significant for ensuring coating stability, promoting good cell response (such as uniform spreading), reducing the risk of local corrosion, and potential friction and wear issues. Additionally, as shown in Figure 1K, we performed XRD analysis on the Mg-Ca-nHAC composite scaffolds. The spectrum revealed the constituents of Mg(OH)_2_ and hydroxyapatite structures. Furthermore, to verify the degradation products of Mg alloy scaffolds, Figure 2E shows that a large number of gas bubbles were generated from Mg alloy composite scaffolds in a liquid environment. To further identify the main components of these bubbles, the gas was collected for gas chromatography analysis. As shown in Figure 2F–H, it was found that the amount of hydrogen generated by Mg alloy composite scaffolds increased with the prolongation of time and it was more favorable to promote the hydrogen in the acidic environment. It is well known that tumors are weakly acidic environments [55], so we speculated that the Mg alloy composite scaffolds may further exert anti-tumor effects by promoting the generation of hydrogen. The porosity of Mg-Ca-nHAC composite scaffolds was determined using mercury porosimetry. As shown in Supplementary Information Figure S1, the porosity was 4.5081%.

Extracellular acidity may influence cancer cells by promoting dedifferentiation, inducing EMT, and enhancing stem-like properties [56]. Therefore, it is shown that targeting acidity can improve tumor response. One treatment strategy may be to alkalize the tumor microenvironment using a whole-body buffer such as sodium bicarbonate (sodium bicarbonate). H_2_ has antioxidant properties [57], and it can regulate the expression of tumor-associated ROS genes by selectively reducing the concentration of hydroxyl radicals, the most potent oxidant known in humans [58]. H_2_ can reduce ROS, inhibit vascular endothelial growth factor (VEGF), down-regulate ERK signaling pathway [59], and it also activates the body’s own antioxidant system by up-regulating the production of endogenous antioxidants, such as glutathione, catalase and superoxide dismutase [60, 61]. ROS can be generated in a variety of biological processes, including mitochondrial respiratory chain reactions, inflammatory responses and oxidative stress due to exposure to environmental toxins and radiation [62]. As a result, ROS has emerged as a potential target for cancer therapy and has given rise to two therapeutic strategies: antioxidant therapy and pro-oxidant therapy. While antioxidant therapy restores the dynamic balance of the microenvironment by removing excess ROS, pro-oxidant therapy aims to increase the level of ROS in cancer cells in order to induce cell death [63]. As shown in Figure 4F, ROS assay was performed in this study and in HeLa cells, ROS levels in the Mg-Ca and Mg-Ca-nHAC composite groups were significantly and statistically increased compared to the control group, especially in the Mg-Ca-nHAC composite group (**P < 0.01). In SiHa cells, ROS levels were also significantly increased in the Mg-Ca-nHAC composite group (**P < 0.01). Therefore, we can hypothesize that Mg-based composite scaffolds are treated by pro-oxidative therapy to increase ROS levels in cancer cells to induce cell death. In addition, high levels of ROS can damage mitochondrial DNA, protein and lipids, further disrupting mitochondrial function and leading to a further decrease in mitochondrial membrane potential [64]. This vicious cycle may lead to cell damage and even cell death. In Figure 3H, we used JC-1 fluorescent dye labeling to detect changes in mitochondrial membrane potential. The experimental results showed that the mitochondrial membrane potential was significantly decreased in SiHa cells, especially in the Mg-Ca-nHAC composite scaffolds group. In addition, we tested the main degradation products of composite scaffolds, Mg^2+^ and Ca^2+^, and the results of the study showed that Mg^2+^ changed with pH, and the concentration of Mg^2+^ increased significantly in the acidic environment. This result suggested that the acidic microenvironment of the tumor was conducive to the release of Mg^2+^, which in turn exerted anti-tumor effects. Mg^2+^ played a bidirectional role in cancer development, with the role of Mg^2+^ changing dynamically at different stages of cancer development. Mg^2+^ participated in a variety of biochemical reactions that regulate cellular functions, including inhibition of chemical carcinogenesis, DNA replication and repair, apoptosis, proliferation, metastasis, reduction of inflammation and oxidative stress [65, 66]. Thus, the degradation of Mg mainly produces high concentrations of Mg^2+^, which can be used as a local implant material for tumor therapy. To determine whether the Mg alloy scaffolds possess anti-tumor effects, we therefore first evaluated their influence on cell proliferation via the CCK-8 assay. As shown in the results of the study in Figure 3B–D, both Mg-Ca and Mg-Ca-nHAC composite scaffolds significantly inhibited the growth of cervical cancer cells, and SiHa cells were the most sensitive to the effect of the treated group, and the inhibitory effect on SiHa cells was stronger than that on HeLa cells. It is possible that the corrosion product Mg^2+^ inhibited tumor progression mainly by inhibiting tumor cell proliferation, promoting apoptosis, inhibiting migration and invasion and ameliorating the tumor inflammatory response. The prerequisite for cell proliferation was cell adhesion [67].

To verify the effect of Mg-Ca-nHAC composite scaffolds on cell adhesion, this study employed phalloidin fluorescence staining and quantitative analysis of fluorescence intensity. The results are shown in Figure 3E–G: in the SiHa cells, especially the cytoskeleton of the Mg-Ca, Mg-Ca-nHAC group failed to stretch obviously, the cell adhesion was relatively poor, and there were obvious differences with control group difference with statistical significance (**P < 0.01). It was also shown that Mg-Ca scaffolds and Mg-Ca-nHAC composite scaffolds impaired cell adhesion and, further, cell proliferation—with the Mg-Ca-nHAC composite scaffold group exhibiting the most pronounced effect. In order to further verify the effect of Mg-Ca-nHAC composite scaffolds on the proliferation and growth of cervical cancer, this study carried out live-dead cell staining experimental method to verify and quantitatively analyze the fluorescence intensity. As shown in Figure 4A and B, live-dead cell staining revealed that the Mg-Ca-nHAC composite scaffolds group exhibited the highest level of red fluorescence and the lowest level of green fluorescence, indicating the greatest number of dead cells, followed by the Mg-Ca and nHAC groups. It indicates that all treatment groups have the effect of inhibiting cervical cancer, especially that the Mg-Ca-nHAC composite scaffold has the most obvious inhibitory effect. This result is consistent with the results of CCK8 experiment. Figure 4C and D shows the effect of different treatment groups on the HeLa cell cycle detected by flow assay, and the results showed that nHAC, Mg-Ca-nHAC composite scaffolds inhibited the cell growth mainly in the G2/M phase, that is, in the late cytokinesis and interphase. The effect of different treatment groups on SiHa cell cycle was detected by flow assay as shown in Figure 4C, and the results indicated that the nHAC group inhibited cell growth mainly in the G2/M phase, that is, in the late and interphase of cell division. nHAC and Mg-Ca-nHAC composite scaffolds groups inhibited cell growth mainly in the S phase, that is, in the phase of cell replication, apoptosis and proliferation were closely coupled, and inadequate apoptosis can lead to uncontrolled cell proliferation. For more than three decades, a major goal and objective of clinical oncology has been the development of treatments that promoted the effective elimination of cancer cells through apoptosis [68]. Cancer cells are stained with FITC and PI-labeled fluorescent dyes for the membrane-bound protein V. Subsequent observation revealed significant apoptotic structural changes, namely nuclear fragmentation and chromatin condensation, which are hallmark features of apoptosis [69]. In addition, flow cytometry was used to detect cell apoptosis. Results showed that the Mg-Ca-nHAC composite scaffold group exhibited the most obvious inhibition of cell growth, with significant differences between groups (*P < 0.05, **P < 0.01), as shown in Figure 4E. In order to study the effect of degradable Mg-based materials on tumor metastasis, cell migration and invasion were analyzed. The experimental results are shown in Figure 5A: Compared with the blank group, the Mg-Ca-nHAC composite scaffolds group significantly inhibited cell migration, followed by the Mg-Ca group (*P < 0.05, **P < 0.01). Compared with the nHAC group and the intergroup, there was also a significant inhibitory effect. Transwell assay was used to detect cell invasion, and the experimental results are shown in Figure 5B: compared with the blank group, the Mg-Ca-nHAC composite scaffolds group significantly inhibited cell invasion, followed by the Mg-Ca group (*P < 0.05, **P < 0.01). There was also a significant inhibition when compared with the nHAC group and between groups. The results of the scratch assay were also consistent with those of cell invasion and migration assays, with the Mg-Ca-nHAC composite scaffold group significantly inhibiting cell migration. (as shown in Figure 5C and D). Figure 5E shows that the Mg-Ca-nHAC composite scaffolds were the weakest in cell cloning ability, followed by Mg-Ca group and nHAC group. nHAC is composed of collagen type I and hydroxyapatite. Thus, it is possible that the hydroxyapatite structure promotes the generation of Mg(OH)_2_ precipitates. In addition, Mg(OH)_2_ may inhibit tumor-associated inflammation by suppressing ROS production through inhibition of inflammatory vesicles [14]. In terms of genomic instability and mutation, Mg(OH)_2_ may ameliorate DNA double-strand breaks to inhibit tumor progression [70]. Further, we found that Mg-Ca-nHAC composite scaffolds can promote the proliferation, migration, adhesion and osteogenic differentiation through the classical Wnt pathway (Figure 6). According to Figure 7A, the results showed that Mg-Ca-nHAC composite scaffolds showed significant decrease in Wnt, β-catenin and TCF7 protein expression levels in HeLa/SiHa cells compared to the control group and the other treatment groups (*P < 0.05,**P < 0.01,***P < 0.001). Besides, in the PCR assay (as shown in Figure 7C), the results showed that compared to the control group and other treatment groups, Mg-Ca-nHAC composite scaffolds showed significant decrease in Wnt, β-catenin, TCF7, N-cadherin, Vimentin, Snail, Twist gene expression levels in HeLa/SiHa cells (*P < 0.05,**P < 0.01,***P < 0.001). However, the expression level of E-cadherin in the Mg-Ca-nHAC group showed significant increase. Additionally, this study conducted a reverse validation to further explore the anti-tumor mechanisms of Mg-Ca-nHAC. The experimental results regarding cell viability are shown in Figure 8A. LGK974 and Mg-Ca-nHAC significantly inhibited the viability of HeLa/SiHa cells, and the combination therapy (LGK974+Mg-Ca-nHAC) exhibited a synergistic effect. In terms of cell migration (Figure 8B), LGK974+Mg-Ca-nHAC and Mg-Ca-nHAC significantly inhibited the migration ability (****P < 0.0001). In Western blot and PCR experiments (Figure 8C–F), LGK974, Mg-Ca-nHAC, LGK974+Mg-Ca-nHAC groups significantly inhibited the Wnt/β-catenin/TCF7 pathway in HeLa and SiHa cells (downregulating Wnt, β-catenin and TCF7) and reversed the EMT process (upregulating E-cadherin and inhibiting N-cadherin/Vimentin). In summary, Mg-Ca-nHAC and LGK974 exerted their anti-cervical cancer effects by synergistically inhibiting EMT through the Wnt/β-catenin/TCF7 signaling pathway. The Wnt signaling cascade is a critical regulator of many biological processes, such as embryonic development, cell cycle control, inflammation and tumor development [71]. TCF7 plays a role in a variety of biological processes, including cell proliferation, differentiation, tissue development and tumorigenesis [72]. In the presence of activation of the Wnt/β-catenin signaling pathway, β-catenin accumulated in the nucleus and interacted with transcription factors such as TCF7. This interaction enhanced the binding ability of TCF7 to the promoters of EMT-related gene, thereby promoting the transcription of these gene [73]. For example, β-catenin and TCF7 may co-activate the expression of EMT-inducing factors such as Snail, Slug and Twist, which further promote mesenchymalization of epithelial cells [74]. EMT is known to be a key factor in tumor invasion and metastasis [75]. According to the above results (Figures 7 and 8), Mg-Ca-nHAC composite scaffolds targeting EMT through the Wnt signaling pathway are hotspot for tumor therapy in the future. In other studies, the authors observed reduced cell migration and invasion when the osteosarcoma cell line (U2OS) was co-cultured with Mg-based material extracts. ERK1/2, JNK and p38 were activated through MAPK signaling pathways [76]. This suggests that the promigratory and proinvasive effects of Mg-based materials may be mediated by distinct signaling pathways, such as those involving changes in MMP activity [77]. This may be due to the different degradation products of Mg matrix composites exerting different mechanisms of anti-tumor effects, which need to be further explored in depth in the future.

In this study, an *in vivo* animal model was used to further validate its anti-tumor effects and biosafety. Firstly, we constructed subcutaneous tumor animal model (as shown in Figure 9A and B). In addition, we also used a mouse tumor model carrying the expression of luciferase (HeLa-Luc) to visualize the effect of composite scaffolds treatment with diminished fluorescence intensity on live imaging and the Mg-Ca-nHAC group implantation had the weakest bioluminescence signal and the best tumor growth inhibition, and we observed the changes of the weight of the mice and the volume of the tumors at 0, 7 and 14 days, the biosafety of implanted materials is an important consideration. Therefore, in the present study, post-implantation mice were tested for hematology. As shown in Figure 9C, there was no significant difference in the white blood cell, hemoglobin and platelet counts in the treated group compared to the control group. This indicated that nHAC group and Mg-Ca group were biologically safe and had no significant side effects *in vivo*, which was a prerequisite for their application to the clinic. Furthermore, we observed the weight of mice at different time points and found that there was no significant change in the weight of mice in the treatment group (as shown in Figure 9D). It indicated that Mg-Ca-nHAC group treatment did not cause significant side effects. We then performed tumor sampling at 14 days and found that the Mg-Ca-nHAC group had the smallest tumor weight compared with the other groups, followed by the nHAC group and Mg-Ca group, and there was a statistical difference. It indicated that the Mg-Ca-nHAC group significantly inhibited tumor growth (Figure 9H and I). The results of *in vivo* imaging (Figure 9E–G) were consistent with the results of Figure 9H and I, and the Mg-Ca-nHAC group significantly inhibited tumor growth. HE staining and Tunel staining of tumor sections performed after sampling further confirmed that Mg-Ca-nHAC group implantation induced severe tumor cell apoptosis (Figure 9K). In addition, there were no novital organs such as the lung, liver, spleen, kidney and heart of mice implanted with Mg-Ca-nHAC group, indicating that there were no toxic side effects (Figure 9J). Ki67 is a marker to test the active proliferation of tumor cells [78]. It was found that Mg-Ca-nHAC group had the lowest expression of Ki67, and the tumor cells were not in the stage of active proliferation, which also indicated that Mg-Ca-nHAC group significantly inhibited the growth of cervical cancer. Immunohistochemical staining results were consistent with the *in vitro* experiments (Figure 9L and M). To further validate the mechanisms of action, this study used an *in vivo* murine model. This *in vivo* study demonstrated the potent anti-tumor efficacy and biological safety of LGK974+Mg-Ca-nHAC therapy (Figure 10A). Compared with the control group, all treatment groups showed a significant reduction in tumor volume (Figure 10B) and weight (Figure 10C). HE detection revealed extensive necrosis (>50%) and severe structural disruption in the LGK974+Mg-Ca-nHAC group (Figure 10D). Mg-Ca-nHAC significantly suppressed cell proliferation, as evidenced by decreased Ki67 expression (Figure 10E), and promoted apoptosis (Figure 10F). Furthermore, Mg-Ca-nHAC inhibited the EMT process, which was characterized by upregulation of E-cadherin and downregulation of N-cadherin and Vimentin (Figure 10G–I). Figure 10J–L results indicated that the antitumor effect of Mg-Ca-nHAC was mediated through inhibition of the Wnt/β-catenin/TCF7 pathway. Based on the above experimental results, Mg-Ca-nHAC composite scaffolds target EMT through the Wnt/β-catenin/TCF7 signaling pathway. The inhibitory effect of Mg-Ca-nHAC on the Wnt/β-catenin/TCF7 signaling pathway was superior to that of the inhibitor LGK974, while the synergistic inhibitory effect of LGK974+Mg-Ca-nHAC was the most significant. *In vivo* reverse validation experiments yielded consistent results with *in vitro* reverse experiments, further confirming that Mg-Ca-nHAC composite scaffolds target EMT via the Wnt/β-catenin/TCF7 signaling pathway. This had great potential for clinical application in anti-tumor therapy.

However, different degradation products of Mg matrix composites exert anti-tumor effects by different mechanisms. Research has demonstrated that Mg^2+^ exerts anti-tumor effects by targeting multiple signaling pathways. It suppresses tumor proliferation through mechanisms involving the TRPM7 channel and PPM1D phosphatase, while inducing apoptosis via modulation of the AKT/mTOR and Bax pathways [79]. Furthermore, Mg^2+^ has been shown to inhibit tumor invasion and metastasis by downregulating the TNF-α/IL-1/NF-κB signaling cascade [80]. Additionally, Mg(OH)_2_ contributes to tumor growth suppression by blocking the PI3K/AKT pathway and activating mTORC1, thereby reducing cancer cell proliferation [81]. The uniqueness of the Mg-Ca-nHAC scaffold lies in its integration of the dual advantages of Wnt pathway targeting and traditional Mg^2+^ regulation. The sustained release of Mg^2+^ can inhibit autophagy through TRPM7 and block EMT through the Wnt pathway. Mg^2+^ dependent inhibition of TRPM7 significantly reduced tumor invasiveness, expanding the mechanism of regulation through ROS balance-Mg^2+^ alleviated oxidative damage. Abnormal activation of the Wnt signaling pathway is one of the core mechanisms driving EMT [82]. Although we have demonstrated the excellent anti-tumor effect of Mg-Ca-nHAC composite scaffolds, the underlying mechanisms still need to be investigated in the future. The anti-tumor effects of Mg alloys were mainly due to their degradation products. According to the results of the above studies, it also shows that nHAC had some anti-tumor effects, probably due to the role of its main components, nano-hydroxyapatite and collagen type I. Previous studies have reported that nano-hydroxyapatite exhibits anti-tumor effects [14]. Nano-hydroxyapatite may influence cell signaling by interacting with receptors on the cell surface, thereby regulating tumor cell growth and apoptosis. Nano-hydroxyapatite can inhibit the Wnt pathway by slowly releasing Ca^2+^ [83]. Type I collagen is a major structural protein in the human body that forms a 3D network structure, providing support and attachment sites for cells [84]. In nHAC, type I collagen can enhance the mechanical properties and bioactivity of the material, promote cell adhesion and growth and also help maintain tissue integrity and stability. The cell adhesion molecules and other components bound to it may play an important role in regulating the interaction between tumor cells and the microenvironment [84]. However, due to its insufficient mechanical strength and slow gelation rate, it has not been fully utilized. To address these limitations, Xiao et al proposed a novel dual-network methacrylated collagen-magnesium (CMA-Mg) hydrogel fabricated via digital light processing 3D printing. This significantly enhanced mechanical stability, printability and resistance to enzymatic degradation. CMA-Mg hydrogels exhibited excellent biocompatibility, promoting cell proliferation, migration and differentiation [84]. This study also combines the synergistic effects of Mg-Ca alloys and nHAC composite materials. In addition, type I collagen fibers can regulate the integrin α2β1-Snail1 axis to inhibit EMT [85]. The specific mechanisms of action of nano-hydroxyapatite and type I collagen in nHAC require further investigation to clarify their synergistic or independent roles in the anti-tumor process.

In summary, Mg-Ca alloy and nHAC have better synergistic inhibition of cervical cancer. It also utilizes the advantages of both metal materials and nanomaterials, avoiding the rapid degradation problem of metal materials and the mechanical support problem of nanomaterials, and it has a greater potential in the field of oncology in the future. During the degradation of Mg-based materials, the degradation of OH^−^ and Mg(OH)_2_ produced by the cathodic reaction neutralizes the acid and increases the pH, thus inhibiting tumor progression [86, 87]. These products inhibit tumor progression mainly by modulating the acidic tumor microenvironment, resisting oxidative stress, alleviating tumor inflammation, inhibiting tumor cell proliferation, promoting apoptosis and directly reducing migration and invasion. It also provides new treatment options for the adjuvant treatment of cervical cancer and alleviates the side effects of surgery and radiotherapy.

## Conclusion

According to the results of *in vivo* and *in vitro* studies, Mg-Ca-nHAC composite scaffolds are a promising biomaterial with anticancer activity, which can inhibit cell proliferation, migration and invasion and promote apoptosis. This study also shows that Mg alloy composite scaffolds’ degradation products target EMT through the Wnt/β-catenin/TCF7 signaling pathway, making it have potential anti-tumor properties. In addition, as the composition of Mg-based composite scaffolds changes, whether they exert anti-tumor effects through other signaling pathways needs to be further explored. More importantly, the biosafety of implanted materials is an important consideration. On one hand, the release of degradation products is regulated by modulating the material preparation to achieve better anti-tumor effects. On the other hand, non-specific damage to normal cells is minimized. It provides theoretical and experimental basis for the development of therapeutic adjuvant materials for cervical cancer with good biocompatibility, excellent anti-tumor properties and suitable biodegradability.

## Supplementary Material

rbaf110_Supplementary_Data

## Data Availability

Mendeley Data, V1, doi: 10.17632/m3mypy4t77.1
