# Supplementary material for: Study on the role and mechanism of magnesium-calcium-mineralized collagen composite scaffolds in the adjuvant treatment of cervical cancer
Source: Regen Biomater. 2025 Nov 12;12:rbaf110. doi: 10.1093/rb/rbaf110 (PMC12684717; doi:10.1093/rb/rbaf110)
Supplement: rbaf110_Supplementary_Data [file rbaf110_supplementary_data.zip › rbaf110_Supplementary_Data.zip]

**Supplementary Materials**

Study on the role and mechanism of magnesium-calcium-mineralised collagen composite scaffolds in the adjuvant treatment of cervical cancer

Xiaojing Nie^1,2,3^, Lei Wang^1^, Wang Yang^2^, Muyesier Maimaiti^4^, Jingxin Yang^5*^, Chen Lin^2*^

^1^School of Public Health, Xinjiang Medical University, Urumqi, Xinjiang 830000, P.R.China.

^2^Department of Pathology, School of Basic Medical Sciences, Xinjiang Medical University, Urumqi, Xinjiang 830000, P.R.China.

^3^Institute of Medical Sciences of Xinjiang Medical University, Urumqi, Xinjiang 830000, P.R.China.

^4^Pathology Department of Xinjiang Production and Construction Corps Hospital, Urumqi, Xinjiang 830000, P.R.China.
^5^Beijing Engineering Research Center of Smart Mechanical Innovation Design Service, Beijing Union University, No.4 Gongti North Road, Chaoyang District, Beijing100027,P.R.China.

* Correspondence Authors E-mail: Jingxin Yang [myxin825@163.com](mailto:myxin825@163.com), Chen Lin [linmao1977@126.com](mailto:linmao1977@126.com)

Figure S1 Determine porosity using the mercury porosimetry method.

The pore volume distribution predominantly ranged between 0 and 100 nm. Instrumental analysis revealed a porosity of 4.5081%.
